# Analysis of the Influence of Demographic, Clinical and Physical Factors on the Occurrence of Ocular Complications After Ruthenium-106 and Iodine-125 Brachytherapy as Well as Proton Therapy of Uveal Melanoma

**DOI:** 10.3390/cancers18060944

**Published:** 2026-03-13

**Authors:** Jakub Jarczak, Bożena Romanowska-Dixon, Beata Sas-Korczyńska, Andrzej Sokołowski

**Affiliations:** 1 Department of Ophthalmology and Ocular Oncology, University Hospital, 31-501 Krakow, Poland; romanowskadixonbozena1@gmail.com; 2 Doctoral School of Medical and Health Sciences, Jagiellonian University Medical College, 31-530 Krakow, Poland; 3 Division of Ophthalmology and Ocular Oncology, Department of Ophthalmology, Faculty of Medicine, Jagiellonian University Medical College, 31-501 Krakow, Poland; 4 Department of Oncology, Radiotherapy and Translational Medicine, Medicine Faculty, University of Rzeszów Collegium Medicum, 35-959 Rzeszów, Poland; b.sas.korczynska@gmail.com; 5 Faculty of Management, Media and Technology, Andrzej Frycz Modrzewski Krakow University, 30-705 Krakow, Poland; sokolows@uek.krakow.pl

**Keywords:** uveal melanoma, brachytherapy, plaque radiotherapy, proton therapy, radiation complications, ocular complications, visual acuity, vision deterioration, risk factors

## Abstract

Background: The aim of this study was to evaluate the influence of demographic, clinical and physical factors on the occurrence of ocular complications after ruthenium-106 (Ru-106) brachytherapy, iodine-125 (I-125) brachytherapy and proton therapy of uveal melanoma. Methods: A retrospective analysis of 300 patients’ electronic and paper medical records treated for uveal melanoma at the Department of Ophthalmology and Ocular Oncology, University Hospital in Krakow, Poland, from May 2014 to December 2016 was performed. The created database, which includes numerous parameters characterizing patients, tumors, applied treatments and their effects, with particular emphasis on the occurrence of ocular complications, was subjected to detailed analysis. The influence of selected factors on the occurrence of identified complications was checked by performing a univariable Cox proportional hazards regression analysis, and then the factors that were statistically significant were included in a multivariable Cox proportional hazards regression analysis which gave the final results. Results: Of the 300 patients, 125 (41.67%) were treated with Ru-106 brachytherapy (87 (29%) with CCB plaque and 38 (12.67%) with COB plaque), 102 (34%) with I-125 brachytherapy and 73 (24.33%) with proton therapy. Mean follow-up was 88.63 months (median 89, range: 20–127). The occurrence of cataract was associated with the older age of patients. Maculopathy was associated with female sex, younger age, use of I-125 brachytherapy, tumor location involving the macula and/or optic disc and moderate tumor pigmentation. Diagnosis of systemic hypertension was associated with a lower risk of maculopathy. Retinopathy was associated with younger age, tumor location involving the macula and/or optic disc and the use of I-125 brachytherapy. Optic neuropathy was associated with younger age, greater tumor largest base diameter, tumor location involving the macula and/or optic disc and the use of I-125 brachytherapy. Secondary glaucoma was associated with baseline best corrected visual acuity (BCVA) weaker than 0.5, greater tumor thickness, involvement of the left eye and the use of I-125 brachytherapy. Vitreous hemorrhage was associated with greater tumor thickness, tumor location including the macula and/or optic disc and mushroom-shaped tumor. Conclusions: Our study demonstrated an association between demographic, clinical, and physical factors and the occurrence of ocular complications after radiotherapy for uveal melanoma.

## Simple Summary

The objective of this study, which is a retrospective analysis of 300 patients’ medical records, was to assess the influence of various factors related to the characteristics of patients, tumors and treatment applied on the occurrence of ocular complications after radiotherapy for uveal melanoma. Most often, these complications lead to a significant deterioration of visual acuity in the treated eye. All patients were treated at the Department of Ophthalmology and Ocular Oncology, University Hospital in Krakow, Poland. The most common complications of treatment were found to be: cataract (84.36%), maculopathy (52.67%) and retinopathy (46.67%). The factors that were found to be most frequently associated with the occurrence of complications in the multivariable analysis were: tumor location involving the macula and/or optic disc, the use of iodine-125 brachytherapy, younger age and greater tumor thickness. We believe that the study results may contribute to the improvement in existing, and the invention of new, therapeutic methods.

## 1. Introduction

Uveal melanoma (UM) is the most common primary intraocular malignant tumor in adults, with the highest incidence of 5–10 per million in White patients per year [1,2,3,4]. Treatment involves many methods that can be used separately or in combination. These include surgical methods such as endoresection, exoresection and enucleation; and conservative methods such as brachytherapy (BT), proton therapy (PT), stereotactic radiotherapy (SRT), stereotactic radiosurgery (SRS), transpupillary thermotherapy (TTT) and photodynamic therapy (PDT) [5,6,7,8,9,10]. Ruthenium-106 (Ru-106) brachytherapy, iodine-125 (I-125) brachytherapy and proton therapy are currently some of the most popular methods of treating uveal melanoma with very good effectiveness in achieving local control at the level of 59–98% [11], 76–100% [12] and 95% [13] respectively. A breakthrough moment in the popularization of brachytherapy was the publication of the Collaborative Ocular Melanoma Study (COMS) results, showing similar mortality and metastasis rates compared to enucleation in medium-sized and large UM [14,15,16]. Proton therapy is a newer and more precise method, but at the same time it requires highly specialized equipment and is much more expensive than brachytherapy, which significantly limits its availability [8,13]. Unfortunately, both brachytherapy and proton therapy are often associated with serious ophthalmological complications that can significantly impair visual acuity, even leading to blindness and rarely to the necessity of enucleation [17,18,19,20,21]. Complications result primarily from the fact that we are currently unable to limit the radiation precisely to the tumor cells without irradiation of healthy tissues. Depending mostly on the size and location of the tumor, but also on the type of radiotherapy used, healthy structures such as the macula or the optic disc, which are also critical for sharp vision, are often irradiated to varying degrees, which causes their damage [17,22,23,24,25]. Various studies present varied radiation complications, their frequency and severity. The most common ocular radiation complications include cataract, maculopathy, retinopathy, optic neuropathy, secondary glaucoma (mainly neovascular glaucoma), vitreous hemorrhage, retinal detachment, keratopathy, dry eye, conjunctivitis, scleral necrosis, intraocular inflammation, toxic tumor syndrome, eyeball atrophy and others, with the usual accompanying decrease in visual acuity. The most important risk factors for ocular complications after brachytherapy and proton therapy are considered to be: greater tumor thickness; tumor location close to the macula and optic disc; a greater dose of radiation received by the macula, optic disc and retina; plaque size; and the patient’s age. As in the case of the frequency of complications, for the risk factors of their occurrence the results of available studies also often differ significantly, but the size of the tumor and its location are considered crucial [17,18,19,20,21,22,23,24,25,26,27,28]. However, it should be remembered that the growth of the tumor itself and its damage can also lead to the development of abnormalities such as secondary glaucoma, retinal detachment, and vitreous hemorrhage, which also contribute to the deterioration of eye function. Therefore, often, the deterioration of visual acuity after treatment is not only a result of radiation complications but also the impact of the tumor.

The type of treatment used depends on many factors and the most important of them seems to be the size and location of the tumor, general condition of the patient and the availability of treatment methods. In Europe and Asia, the most commonly used radioactive isotope for brachytherapy of uveal melanoma is Ru-106, which emits beta radiation. Due to the range of this type of radiation, it is suitable for tumors with a thickness not exceeding approx. 5–6 mm. In North America I-125 is dominant, which emits gamma radiation. This type of radiation is also suitable for treating larger tumors, according to some sources, even those over 15 mm thick [8,17,20,26,29]. Patients have access to proton therapy much less frequently, which is primarily due to its high price and the necessary complicated equipment. Due to the Bragg peak phenomenon, proton therapy allows for equal irradiation of the tumor mass, sparing the surrounding healthy tissue to a greater extent than brachytherapy [7,8,26].

The Department of Ophthalmology and Ocular Oncology, University Hospital in Krakow is the largest ocular oncology center in Poland where, every year, approximately 350–400 patients are treated for uveal melanoma. Thanks to the wide range of available therapies, we are able to provide patients with an individual approach when choosing the optimal method based on the factors mentioned above. The Krakow center offers Ru-106 brachytherapy, I-125 brachytherapy, transpupillary thermotherapy, surgical methods, and thanks to cooperation with the Institute of Nuclear Physics Polish Academy of Sciences in Krakow proton therapy as well. The use of as many as three main and world-leading radiation methods for treating uveal melanoma in daily practice, combined with the large number of treated patients, provides excellent opportunities to study the effects of treatment. Despite access to various methods with very high effectiveness in local control, we still struggle with radiation complications leading to the deterioration of visual acuity. This is a particularly big problem for patients who are monocular or have poorer vision in the other eye.

The vast majority of studies we found regarding the issue of ophthalmological complications and vision deterioration after conservative treatment of uveal melanoma usually focus on the analysis of one, and less often two, types of treatment [14,15,19,21,22,24,25,27,28,30,31,32,33,34,35,36,37,38,39,40,41,42,43]. Apart from the literature reviews or meta-analyses, we found very few studies examining at least three treatment methods in terms of complications in a single study [18,20,44,45].

We therefore decided to take advantage of our large patient population and availability of the three world-leading uveal melanoma treatment methods to determine the risk factors for the occurrence of ocular complications after therapy. The main aim of this retrospective study was to assess the influence of various factors related to the characteristics of patients, tumors and treatment applied on the occurrence of ocular complications after Ru-106 brachytherapy, I-125 brachytherapy and proton therapy of uveal melanoma. We believe that the obtained results may contribute to the better optimization of treatment by reducing the frequency of complications while maintaining or improving local control.

## 2. Materials and Methods

### 2.1. Patients

The study included 300 patients treated for uveal melanoma at the Department of Ophthalmology and Ocular Oncology, University Hospital in Krakow, Poland, from May 2014 to December 2016. The study included adult Polish citizens diagnosed with uveal melanoma. All of them were White. The study involved a retrospective analysis of patients’ electronic and paper medical records, which were used to create an electronic database. This database was then subjected to detailed statistical analysis. The study received positive approval from the Bioethics Committee of the Jagiellonian University No. 1072.6120.289.2022 issued on 14 December 2022. Due to the fact that the study was retrospective in nature, involved the analysis of previously created medical records, did not interfere in the treatment process in any way, and did not require additional contact with patients, obtaining informed consent from patients was not required.

The inclusion criteria include: over 18 years of age, diagnosis of uveal melanoma, and minimum 5-year follow-up period after treatment. We would like to emphasize that patients who required enucleation during this 5-year period but attended follow-ups for longer than 5 years after treatment were also included in the study. However, in their case, we assumed the date of enucleation as the date of the last follow-up.

The exclusion criteria include: diagnosis of other ophthalmological diseases in the treated eye, unrelated to the presence of the tumor, e.g., glaucoma; age-related macular degeneration; diagnosis of ophthalmological complications of general diseases, e.g., diabetic retinopathy; condition following an eye injury that impairs vision to any degree; amblyopia; previous treatment of the eye for uveal melanoma or other tumors by any method; and follow-up time less than 5 years after treatment. These restrictive criteria for patient inclusion and exclusion were intended to exclude, to the greatest extent possible, the influence of factors other than the stated demographic, clinical and physical factors on the occurrence of ocular complications in the treated eye. However, it should be emphasized that 102 patients with diagnosed cataract, and 19 patients who had undergone cataract removal and implantation of an artificial intraocular lens (IOL), were included in the study. We decided not to exclude these patients because with many of them, this was an initial cataract, which often had little or no impact on visual acuity, and it is a common finding in the age group of patients with uveal melanoma. Also, cataract may be the result of the presence of a tumor. At the same time, we excluded these patients from the analysis for cataract as a complication of radiotherapy. Also, 78 patients with retinal detachment, which, at the base of the tumor, is one of the characteristic features of uveal melanoma; and vitreous hemorrhage (5 patients), which often accompanies the diagnosis of uveal melanoma, were included in the study. For this reason, exclusion from the study seems unjustified. We noted their presence before therapy and did not consider them as treatment complications and, similarly to the patients with cataract, we excluded them from the analysis for these specific abnormalities identified as complications after treatment.

Due to the fact that complications following radiotherapy most often become apparent after several years, we assumed a minimum observation period of 5 years [17,25,28]. Our goal was to exclude patients who, due to too short a time after treatment, had not developed ocular complications from radiotherapy because they had died earlier, e.g., due to metastatic disease, or had stopped coming for follow-ups for unknown reasons.

Four patients were additionally excluded because the actual radiation dose absorbed by the ocular structures could not be determined. These were patients with huge tumors who did not agree to enucleation, so an attempt to use I-125 brachytherapy with plaque displacement was made to ensure that the entire tumor mass was sufficiently irradiated.

We also excluded 2 patients treated with a combination of I-125 brachytherapy and TTT as well 13 patients treated with a combination of Ru-106 brachytherapy and TTT commonly called a “sandwich method” [1,7,38,40,46]. We made this decision because their number was too small to create a separate, reliable group for analysis, and the use of TTT in addition to I-125 or Ru-106 brachytherapy meant that their inclusion in the group treated with brachytherapy alone would be inappropriate in our opinion.

In summary, 300 patients were included in the study. Of these, 125 were treated with Ru-106 brachytherapy, 102 with I-125 brachytherapy, and 73 with proton therapy.

### 2.2. Baseline Data

The study included the following patient demographic and clinical characteristics: sex, age, affected eye, comorbidities (diabetes mellitus, systemic hypertension), family history of cancers (parents, grandparents, siblings, children, siblings of parents), best corrected visual acuity (BCVA), and co-existing ocular diseases and symptoms which were not excluded from the study (cataract, implanted IOL, retinal detachment, vitreous hemorrhage).

Tumor data included thickness, largest base diameter, shape (flat, domed, mushroom-shaped), pigmentation (heavy, moderate, light or amelanotic), and location (tumors were divided into the following 4 groups based on location: 1. the macula and/or optic disc, which include tumors involving the macula, optic disc or both of them; 2. posterior to the equator without the macula and optic disc; 3. the equator; 4. anterior to the equator, which include tumors of the iris, iris and ciliary body, ciliary body, ciliary body and choroid, and other choroidal tumors located anterior to the equator). Tumor size measurements were performed using ultrasonography (USG) and/or ultrasound biomicroscopy (UBM). Based on the above parameters, tumors for which it was possible were assigned to T-category staging according to the 8th edition of American Joint Committee on Cancer (AJCC) classification and COMS classification [14,15,16,47,48]. We considered the specific treatment of iris tumors in the AJCC classification, and all those found in our study were classified as T1 category. The COMS classification has changed over the years and slightly different variants can be found in the literature, so here is the specific size division we used: small: height < 2.5 mm and largest base diameter 5–16 mm; medium: height 2.5–10 mm and largest base diameter < or =16 mm; large: height > 10 mm or largest base diameter > 16 mm. We would like to point out that, due to the specificity of the COMS classification regarding tumor location, we did not include tumors arising from the iris and ciliary body in the case of this classification.

### 2.3. Examination

Patients were examined and treated by doctors, nurses, and support staff working in the hospital, but it should be emphasized that these were often different people over the years. The same applies to equipment used, which has changed over time but has always met all the required criteria.

First-time and follow-up examinations included BCVA examination in the form of Snellen visual acuity measurement as a decimal value except for the weakest values (1.0, 0.9, 0.8, 0.7, 0.6, 0.5, 0.4, 0.3, 0.2, 0.1, 0.05, counting fingers, hand motions, light perception, no light perception). Patients who underwent enucleation from the day of its performance were treated as patients in the lowest BCVA group with no light perception, as in Shields et al.’s study [20]. Based on these results, patients were assigned to one of the three BCVA groups for most of the further analysis: ≥0.5; <0.5 and >0.1; ≤0.1.

The following tests were also performed: full slit lamp examination, intraocular pressure using the non-contact method, USG, optical coherence tomography (OCT) of the macula, photography of the tumor and also if necessary intraocular pressure using an applanation method, OCT of the optic disc and tumor, UBM, gonioscopy or other tests as needed. Each time, an interview was conducted regarding symptoms, ailments, general and ophthalmological diseases, and also, during the follow-up, the results of laboratory tests (liver enzymes), imaging tests (chest X-ray or computed tomography, abdominal ultrasound or computed tomography) and any other tests performed under the supervision of the attending oncologist to search for metastasis.

Follow-up examinations were carried out usually after 6 months (with the first assessment of the tumor’s response to treatment), 1 year, 2 years, 3 years after treatment, and then every 2 years. In some cases, if recurrence was suspected, complications occurred, or for other reasons, the frequency of follow-up visits could be increased. Patients also sometimes postponed scheduled visits for various reasons (e.g., due to another illness) or requested an earlier visit due to concerns about a possible recurrence or complications. Rarely, they simply missed some of their follow-up examinations. During follow-up examinations, apart from the tumor status, special attention was paid to the occurrence and time of appearance of potential complications, which include, among others: maculopathy (defined as macular edema or atrophy seen clinically and/or on OCT), retinopathy (defined as retinal neovascularization, hemorrhages, microaneurysms, hard exudates, or cotton wool spots), optic neuropathy (defined as optic nerve edema or pallor), cataract, secondary glaucoma (defined as intraocular pressure > 21 mmHg and in the case of neovascular glaucoma with neovascularization of the iris or angle), vitreous hemorrhage, hyphema, retinal detachment, keratopathy, dry eye, scleral necrosis, and eyeball atrophy.

### 2.4. Treatment

The time from diagnosis to the start of treatment ranged from several days to several weeks and, due to easier availability and less preparation, it was significantly shorter in the case of brachytherapy than proton therapy. Patients were treated with 3 main methods, which were selected based on the characteristics of the tumor, patient’s condition and taking into account the limitations of the ruthenium and iodine plaques resulting from the radiation range as described above. Proton therapy, as the most precise method, was intended especially for patients whose tumor was located in the posterior pole, close to the structures critical for vision, which are the optic disc and macula, and provided that the patient’s condition enabled their cooperation during the therapy. In the case of tumors where more than one method could be used, the patient was allowed to choose one after being familiarized with the specifics of the proposed therapies. A total of 125 patients (41.67%) were treated with Ru-106 brachytherapy, 102 patients (34%) with I-125 brachytherapy, and 73 patients (24.33%) with proton therapy. For Ru-106 brachytherapy, 2 types of plaques (applicators) were used depending on the location and size of the tumor (Eckert & Ziegler BEBIG, Berlin, Germany; types: CCB—round, diameter 20.2 mm and COB—notched, diameter 19.8 mm). CCB plaque was used in 87 patients (29%) and COB plaque in 38 patients (12.67%). COB plaque is dedicated to tumors adjacent to the optic disc but due to its smaller size than CCB plaque, it has also been used to treat small tumors in other locations as well. In order to analyze the treatment used, we decided to divide patients treated with ruthenium radiotherapy into those treated with CCB plaque and those treated with COB plaque.

For I-125 brachytherapy, a round applicator with a diameter of 20.5 mm (POLATOM, Otwock, Poland) with iodine seeds (Eckert & Ziegler BEBIG, Berlin, Germany) was used.

Brachytherapy plans were prepared by a team of oncology radiotherapists and medical physicists working at the Department of Ophthalmology and Ocular Oncology, University Hospital in Krakow, Poland, using the treatment planning system (Plaque Simulator 5.3.9, BEBIG, Berlin, Germany). The treatment protocol included the primary goal of providing a dose of 80–120 grays (Gy) to the tumor apex and at least 200 Gy to sclera if possible. The applicator was sutured and removed using standard surgical techniques, with the possibility of temporarily severing the extraocular muscles if necessary. The applicator was sutured onto the sclera at the base of the tumor, visualized using transillumination and maintaining a minimum margin of about 2 mm. The applicator suturing and removal procedures were performed under local anesthesia and sedoanalgesia. It should be emphasized that if the tumor base was significantly smaller than the diameter of the plaque and located near the macula or optic disc, the applicator was sutured decentered as far away from the posterior pole as possible to minimize irradiation of structures critical for vision, but maintaining a minimum margin of about 2 mm.

Proton therapy was performed at the Institute of Nuclear Physics Polish Academy of Sciences in Krakow, initially using an isochronous cyclotron AIC-144 (Institute of Nuclear Physics Polish Academy of Sciences, Krakow, Poland) for patients treated in 2014–2015, and later in 2016 using the isochronous cyclotron Proteus C-235 (Ion Beam Applications, Louvain-la-Neuve, Belgium). The treatment plan was prepared using the Eclipse Ocular Proton Planning system, version 13.5.01 (Varian Medical Systems, Palo Alto, CA, USA). Patients were treated according to the 60 CGE (Cobalt Gray Equivalent) dose protocol given in 4 doses of 15 CGE each over 4 days. Proton therapy was preceded by a surgical procedure to place 4 tantalum markers (Altomed, Boldon Colliery, UK) on the sclera as well as numerous imaging tests and preparations.

In summary, Ru-106 brachytherapy was used for tumors that were no more than approximately 6 mm thick. Ru-106 COB plaque, thanks to its notched shape, was used especially for tumors adjacent to the optic disc but due to its smaller size than Ru-106 CCB plaque it has also been used to treat small tumors in other locations as well. I-125 brachytherapy was used for larger tumors that were greater than approximately 6 mm in thickness but no larger than approximately 15 mm. Proton therapy, as the most precise method, was intended especially for patients whose tumor was located in the posterior pole, close to the structures critical for vision, which are the optic disc and macula, in order to minimize the irradiation of these structures. It was also chosen when the location of the tumor in the posterior pole made it impossible for technical reasons to precisely place the radioactive isotope plaque in this area. In order for patients to undergo proton therapy, their general condition had to allow them to cooperate with the staff performing the therapy, which involved, among other things, looking in a given direction, keeping their body in a specific position and following other instructions. In the case of brachytherapy, the patient’s ability to cooperate was not that important. In the case of tumors where more than one method could be used, the patient was allowed to choose one after being familiarized with the specifics of the proposed therapies.

The dates of failure of primary treatment and tumor recurrence, as well as their treatment methods, were recorded. However, they were not subjected to detailed analysis, as this is not the aim of this study. Retreatment methods for primary treatment failure included: Ru-106 brachytherapy, I-125 brachytherapy, TTT, and enucleation. In patients who required additional treatment due to lack of a positive tumor response to primary treatment or subsequent recurrence, only the primary treatment method was taken into account to assess the impact of the treatment method on the occurrence of ocular radiation complications.

Once treatment complications were identified, if medically possible all patients were given the opportunity to have them treated at the Department of Ophthalmology and Ocular Oncology, University Hospital in Krakow. The therapies offered after complications were identified included: anti-vascular endothelial growth factor (anti-VEGF) injections; triamcinolone acetonide (TCA) injections; cataract surgery; antiglaucoma procedures, e.g., trabeculectomy; retinal laser therapy; vitrectomy; phacovitrectomy; and enucleation. Prophylactic treatment with anti-VEGF injections after radiotherapy was not offered. Not all patients who were offered additional treatment for complications decided to do so. Some people decided to undergo treatment for complications, but in centers closer to their place of residence, mainly due to the large distance from Krakow. Information about procedures performed elsewhere was collected during the next follow-up visit. In this study, we did not analyze the efficiency of the treatment of complications and their impact on visual acuity.

Follow-up time was calculated in months from the first day of treatment to enucleation or last visit. Due to formal difficulties in obtaining information about possible death and its cause, we did not analyze mortality in this study.

### 2.5. Statistical Analysis

Data expressed on a qualitative scale were presented as a number and percentage. The Chi-squared test was used to compare the distributions of these parameters between groups. Quantitative data were presented as mean, standard deviation (SD), median and range. Evaluation of the consistency of the distribution of the variables with a normal distribution was performed using the Shapiro–Wilk test. Differences between the groups were analyzed with a Kruskal–Wallis test (followed by a Dunn’s post hoc test). Statistical analysis was performed using Statistica software (v13.3, StatSoft, Tulsa, OK, USA). Statistical analyses were also performed using R software, version 4.5.2 (R Foundation for Statistical Computing, Vienna, Austria), with the use of the survival package (version 3.8-3) for Cox proportional hazards regression. Results were considered statistically significant when *p* < 0.05.

## 3. Results

### 3.1. Baseline Characteristics

A summary of the baseline characteristics of the 300 enrolled patients and tumors is presented in Table 1 and Table 2. Mean age at the first day of treatment was 58.5 years (median 60 years, range: 22–85 years). The diagnosis of uveal melanoma was most often made over a dozen days earlier. A total of 169 patients (56.33%) were women and 131 (43.67%) were men. Mean tumor thickness was 4.73 mm (median 3.53 mm, range: 0.77–13.4 mm) and mean largest base diameter was 11.06 mm (median 10.7 mm, range: 3.17–18.8 mm). Patients were treated with the following methods: ruthenium-106 brachytherapy with CCB plaque (Ru-106 CCB brachytherapy) (87 patients, 29%), ruthenium-106 brachytherapy with COB plaque (Ru-106 COB brachytherapy) (38 patients, 12.67%), I-125 brachytherapy (102 patients, 34%) and proton therapy (73 patients, 24.33%). The mean dose to the apex of the tumor in brachytherapy was 97.33 Gy (median 95.58, range: 73.06–123.4 Gy). Patients who underwent proton therapy were treated according to the protocol of 60 CGE in 4 doses of 15 CGE each over 4 days. Part of our study described in this article did not include analysis of radiation doses.

### 3.2. Treatment Outcomes

The mean follow-up time was 88.63 months (median 89 months, range 20–127 months) (Table 1). A good response to treatment was recorded at the first follow-up visit in 293 patients (97.67%) and further tumor growth in seven patients (2.33%). During follow-up, 23 tumor recurrences were observed (7.67%). In summary, local control was achieved in 272 patients (90.67%) during the follow-up period. The local tumor control rate was 88.51% in Ru-106 CCB brachytherapy, 89.47% in Ru-106 COB brachytherapy, 91.18% in I-125 brachytherapy and 93.15% in proton therapy. There was no statistically significant difference between the different treatment methods in terms of a positive treatment response at the first follow-up, subsequent recurrences and overall local control over the follow-up period (Table 3). Twenty eyes (6.67%) had to be enucleated, of which six eyes were due to the failure of conservative treatment and 14 eyes due to radiation complications. The mean time to enucleation for all patients was 43.60 months (median 32.50, range 13–92). Due to treatment failure and radiation complications, it was 52 months (median: 53, range: 22–92) and 40 months (median: 29.50, range: 13–85) respectively. Each patient who underwent enucleation due to complications most often experienced several complications. The most common complication among these patients was secondary glaucoma (11 patients, 3.67%). More than half of the patients were also diagnosed with vitreous hemorrhage, cataract, and hyphema. Less frequently we observed retinal detachment, retinopathy, maculopathy, and keratopathy. Of the 14 patients who underwent enucleation due to complications, the BCVA immediately before enucleation was: no light perception in 11 patients, light perception in two patients, and hand motions in one patient. In more detail, among patients treated with Ru-106 CCB brachytherapy, enucleation was necessary in five patients (5.75%) (four due to treatment failure and one due to complications); among patients treated with Ru-106 COB brachytherapy, enucleation was not necessary; among patients treated with I-125 brachytherapy, enucleation was necessary in 12 patients (11.76%) (two due to treatment failure and 10 due to complications); and among patients treated with proton therapy, enucleation was necessary in three patients (4.11%) (none due to treatment failure and three due to complications). The presence of distant metastasis was noted in 13 patients (4.33%), and their most common location was the liver (10 patients, 76.92%).

### 3.3. Visual Acuity

During the follow-up, a significant deterioration of visual acuity was observed among treated patients. BCVA better than or equal to 0.5 was present in 182 patients (60.66%) at baseline and 63 patients (21%) at last follow-up. Before treatment, 70 patients (23.33%) had vision worse than 0.5 but better than 0.1, and at the last follow-up 34 patients (11.34%) were in this range. Before treatment, 48 patients (16%) had vision of 0.1 or worse, and at the last follow-up, 203 patients (67.67%) belonged to this group, including 20 patients after enucleation who were classified as having no light perception, which is the method used by Shields et al. [20]. A more detailed distribution of BCVA before and after treatment is presented in Table 4.

### 3.4. Complications

The main aim of this study was to identify factors that may influence the occurrence of ocular complications. This also involved determining their frequency. All complications identified after treatment and their frequency are presented in Table 5. We would like to remind you that we excluded from the analysis into the occurrence of cataract 102 patients in whom it was diagnosed before radiotherapy treatment and 19 patients who had already undergone cataract surgery in the past. Similarly, 78 patients with retinal detachment and five with vitreous hemorrhage before treatment were excluded from analyses for these complications. For the analysis of other complications, all 300 patients were analyzed. The most common complication was cataract, diagnosed in 151 of 179 analyzed patients during the observation period, which is 84.36%. The second most common complication was maculopathy, found in 158 patients, which is an even larger number of patients than in the case of cataract, but due to the larger size of the analyzed group, this means a frequency of 52.67%. The following complications have also been diagnosed less frequently: retinopathy—140 patients (46.67%), optic neuropathy—88 patients (29.33%), secondary glaucoma—83 patients (27.67%), vitreous hemorrhage—56 patients (18.98%), hyphema—25 patients (8.33%), retinal detachment—17 patients (7.66%), keratopathy—7 patients (2.33%), and dry eye—4 patients (1.33%). With the exception of cataract and dry eye, the incidence of ocular complications differed significantly between groups treated with different methods.

To find risk factors for the development of ocular radiation complications, we selected the following 12 demographic, clinical and physical factors for further analysis: age and sex of the patient, diagnosis of systemic hypertension and diabetes mellitus, affected eye, baseline BCVA, tumor location, thickness, largest base diameter, pigmentation and shape, and treatment method. We selected these parameters based on our clinical experience and observations, as well as previous work by other researchers on this topic [14,15,18,19,20,21,22,24,25,27,28,30,31,32,33,34,35,36,37,38,39,40,41,42,43,49,50]. Univariable Cox proportional hazards regression analyses of the influence of selected factors on the occurrence of ocular complications are presented in Table 6 (parts 1 and 2). Then, only factors that were found to be statistically significant in the univariable analysis were used in the multivariable Cox proportional hazards regression analyses (Table 7). Due to the insufficient number of identified cases of hyphema, retinal detachment, keratopathy and dry eye, we refrained from performing univariable and multivariable analyses for these complications. Below we present the outcomes of each ocular complication that occurred during the follow-up period with particular emphasis on the results of multivariable analyses for factors that increased the likelihood of their occurrence.

#### 3.4.1. Cataract

Cataract was diagnosed in 151 patients (84.36%) among 179 analyzed patients during follow-up (Table 5). The mean time to diagnosis of this complication was 28.81 months (median 23, range: 1–106). Cataract was most common among patients treated with I-125 brachytherapy (53 patients, 92.98%) and least common among patients treated with proton therapy (41 patients, 75.93%) (Table 5). On multivariable analysis, only older age was found to be statistically significant for a higher risk of cataract development. It is worth noting that the association with a higher risk of cataract among patients with a baseline BCVA ≤ 0.1 compared with patients with a baseline BCVA ≥ 0.5 reached borderline statistical significance (Table 7).

#### 3.4.2. Maculopathy

A total of 158 patients (52.67%) developed maculopathy during follow-up (Table 5). The mean time to diagnosis of this complication was 35.18 months (median 28, range 3–104). Maculopathy was most common among patients treated with proton therapy (52 patients, 71.23%) and least common among patients treated with Ru-106 COB brachytherapy (10 patients, 26.32%) (Table 5). On multivariable analysis, female sex, younger age and moderately pigmented tumors compared with heavy-pigmented tumors were associated with an increased risk of maculopathy. Interestingly, the presence of systemic hypertension was associated with a lower risk of developing this complication. Location also proved to be statistically significant. Patients with tumors involving the macula and/or optic disc were much more likely to develop maculopathy than those with tumors located at the equator or anterior to the equator. I-125 brachytherapy was found to be associated with a higher risk of maculopathy than Ru-106 COB brachytherapy (Table 7).

#### 3.4.3. Retinopathy

Retinopathy was diagnosed in 140 patients (46.67%) during follow-up (Table 5). The mean time to diagnosis was 40.21 months (median 36.50, range 3–121). Retinopathy was most common among patients treated with proton therapy (43 patients, 58.9%) and least common among patients treated with Ru-106 COB brachytherapy (3 patients, 7.89%) (Table 5). In multivariable analysis, younger age was found to be statistically significant for a higher risk of retinopathy development. Ru-106 COB brachytherapy was associated with an evidently reduced risk of retinopathy compared to I-125 brachytherapy. Tumors located anterior to the equator were associated with a substantially lower risk of this complication in comparison to tumors involving the macula and/or optic disc. The presence of systemic hypertension was found to be of borderline statistical significance as a factor preventing the development of retinopathy. Light-pigmented or amelanotic tumor was found to be of borderline statistical significance as a factor favoring development of this complication compared to heavy-pigmented tumor (Table 7).

#### 3.4.4. Optic Neuropathy

A total of 88 patients (29.33%) developed optic neuropathy during follow-up (Table 5). The mean time to diagnosis of this complication was 40.83 months (median 38, range 3–119). Optic neuropathy was most common among patients treated with I-125 brachytherapy (47 patients, 46.08%) and least common among patients treated with Ru-106 COB brachytherapy (1 patient, 2.63%) (Table 5). In multivariable analysis, older age was associated with a lower risk of developing an analyzed complication. The use of I-125 brachytherapy was associated with a significantly higher risk of optic neuropathy than the use of Ru-106 brachytherapy with CCB and COB plaques. An increase in the tumor’s largest base diameter was associated with a higher risk of developing this complication. Tumor location within the equator of the eyeball and anterior to it was associated with a decrease in the risk of optic neuropathy compared to locations involving the macula and/or optic disc (Table 7).

#### 3.4.5. Secondary Glaucoma

A total of 83 patients (27.7%) developed secondary glaucoma during follow-up (Table 5). The mean time to diagnosis of this complication was 33.43 months (median 27, range: 2–94). Secondary glaucoma was most common among patients treated with I-125 brachytherapy (55 patients, 53.92%) and least common among patients treated with Ru-106 COB brachytherapy (2 patients, 5.26%) (Table 5). In multivariable analysis, a higher risk of developing secondary glaucoma was associated with baseline BCVA weaker than 0.5, greater tumor thickness, and also, interestingly, involvement of the left eye. The use of I-125 brachytherapy was also associated with a significantly higher risk compared to Ru-106 CCB brachytherapy (Table 7).

#### 3.4.6. Vitreous Hemorrhage

Of the 295 patients analyzed, 56 (19%) were diagnosed with vitreous hemorrhage during the follow-up (Table 5). The mean time to diagnosis of this complication was 37.64 months (median 31, range: 4–95). Vitreous hemorrhage was most common among patients treated with I-125 brachytherapy (28 patients, 28.87%) and least common among patients treated with Ru-106 COB brachytherapy (3 patients, 7.89%) (Table 5). In multivariable analysis, a higher risk of vitreous hemorrhage occurrence was associated with greater tumor thickness, tumor location including the macula and/or optic disc compared to all other locations, and mushroom-shaped tumor compared to domed shape (Table 7).

#### 3.4.7. Hyphema

During the follow-up period, anterior chamber hemorrhage was diagnosed in 25 patients (8.3%) (Table 5). The mean time to diagnosis of this complication was 42.36 months (median 38, range: 4–95). Hyphema was most common among patients treated with I-125 brachytherapy (17 patients, 16.67%) and least common among patients treated with Ru-106 CCB brachytherapy (1 patient, 1.15%) (Table 5). Due to the small number of recorded cases, univariable and multivariable analyses were not performed because their credibility would be low.

#### 3.4.8. Retinal Detachment

Of the 222 patients analyzed, 17 (7.7%) were diagnosed with this complication during the follow-up (Table 5). The mean time to diagnosis was 29.82 months (median 18, range: 3–102). Retinal detachment was most common among patients treated with I-125 brachytherapy (8 patients, 17.39%) and least common among patients treated with Ru-106 COB brachytherapy (1 patient, 2.7%) (Table 5). Due to the small number of recorded cases, univariable and multivariable analyses were not performed because their credibility would be low.

#### 3.4.9. Keratopathy

Only seven patients (2.3%) developed keratopathy during follow-up (Table 5). The mean time to diagnosis was 47.29 months (median 46, range: 20–81). Keratopathy was most common among patients treated with I-125 brachytherapy (6 patients, 5.88%) and least common among patients treated with Ru-106 brachytherapy with CCB and COB plaques, in which no patient was diagnosed with this complication (Table 5). Due to the small number of recorded cases, univariable and multivariable analyses were not performed because their credibility would be low.

#### 3.4.10. Dry Eye

Only four patients (1.3%) developed dry eye during follow-up (Table 5). The mean time to diagnosis was 29.25 months (median 15, range: 3–84). Dry eye was most common among patients treated with proton therapy (2 patients, 2.74%) and least common among patients treated with Ru-106 brachytherapy with CCB and COB plaques, in which no patient was diagnosed with this complication (Table 5). Due to the small number of recorded cases, univariable and multivariable analyses were not performed because their credibility would be low.

#### 3.4.11. Summary of Number of Complications

Table 8 shows the total number of complications diagnosed in individual patients. This number varied greatly, from 36 patients (12%) in whom no complications of radiotherapy were observed throughout the entire observation period to as many as 8 complications in 2 patients (0.67%).

### 3.5. Treatment of Complications of Radiotherapy

Complications were treated in a total of 164 patients (54.67%), which represents 62.12% of all patients in whom complications were diagnosed. The following procedures were performed: cataract surgery (94 patients); anti-VEGF injections (91 patients); TCA injections (10 patients); antiglaucoma procedures, e.g., trabeculectomy (12 patients); retinal laser therapy (3 patients); vitrectomy (12 patients); phacovitrectomy (3 patients); and enucleation (14 patients). In this study, we did not analyze the effectiveness and outcomes of treatment of complications.

### 3.6. Treatment of Failures of Radiotherapy

Treatment failures were noted in 28 patients (9.33%). Due to the lack of effectiveness of primary radiotherapy or subsequent tumor recurrence, the following methods were used: Ru-106 brachytherapy (16 patients), TTT (10 patients), I-125 brachytherapy (9 patients), and enucleation (6 patients). The higher number of retreatments than the number of patients with failures is due to the fact that some patients received a combination of two methods, such as Ru-106 brachytherapy combined with TTT, and some had to repeat the treatment several times due to subsequent recurrences.

## 4. Discussion

### 4.1. Planning the Study

Planning our study, we wanted to take advantage of the unique availability of various world-leading treatment methods for uveal melanoma in a single center—the Department of Ophthalmology and Ocular Oncology, University Hospital in Krakow, Poland. Thanks to this, it was possible to include the treatment method in the analyses as one of the factors potentially increasing the risk of radiation complications, while keeping in mind the lack of randomization and differences in the characteristics of patients and tumors treated with different methods. Shields et al. and Aziz et al. analyzed the influence of treatment methods in their studies in a similar way [20,43]. We also wanted to check whether our clinical observations regarding the occurrence of complications after radiotherapy would be reflected in the analytical results and how they compare to the results of other researchers.

When planning our study, we faced several dilemmas. The first dilemma was defining patient inclusion and exclusion criteria. We decided that, since the primary goal of the study is to assess the influence of selected factors on the development of radiation complications that lead to deterioration in visual acuity, these criteria must be restrictive in order to minimize the influence of other confounding factors on the obtained results. We chose a minimum 5-year follow-up period because our experience and the results of other researchers show that radiation complications often appear several years after treatment [19,25]. In a minority of studies on radiation complications and the deterioration of visual acuity after radiotherapy for uveal melanoma, we found information about patients included in the study with ocular comorbidities such as age-related macular degeneration, glaucoma or diabetic retinopathy diagnosed before the primary therapy for the tumor [22,25,28,30,39,43]. The vast majority of studies we have reviewed do not provide any information about their inclusion or exclusion from the studies [18,19,21,24,27,31,35,36,38,40,41,42]. We excluded all patients with ocular diseases that could in any way affect the assessment of complications or the patient’s visual acuity, making an exception only for those in whom the abnormalities found were related to the tumor (retinal detachment, vitreous hemorrhage) as well as patients diagnosed with cataract, which is quite common in the dominant age group of patients diagnosed with uveal melanoma and may also be the result of the presence of a tumor. We believe that our decisions, despite the reduction in the number of patients analyzed, had a positive impact on the reliability of the results.

Further dilemmas concerned the choice of division into categories of the analyzed parameters, the criteria for assessing tumors, defining complications, and others. While analyzing the literature on radiation complications and the deterioration of visual acuity after treatment of uveal melanoma, we encountered very different categories of values in terms of visual acuity; classifications of tumor parameters such as its size, color, shape, and above all, location; as well as definitions of complications. We tried to use in our study the divisions and definitions most frequently used by other researchers to best compare the obtained results with those from other centers. However, we would like to point out that such a huge number of differences in the analyzed parameters between researchers makes a reliable comparison of results, which is difficult in this type of research, even more complicated and not always possible. Uveal melanoma is a rare cancer, and the number of places and specialists who treat it is relatively small. Due to these facts, greater global standardization of the inclusion and exclusion criteria, and classification of parameters and definitions used in scientific research, may be worth considering.

### 4.2. Baseline Characteristics

When analyzing the baseline characteristics of the patients, attention is drawn to the youngest age of patients treated with proton therapy (Table 1). Our observations suggest that this may be due, among other things, to greater concern about maintaining good visual acuity after treatment among young people than among older people. Therefore, when given a choice between proton therapy and brachytherapy, younger patients are more likely to choose proton therapy. An additional factor is the need for a larger number of visits for proton therapy treatment, and the overall duration of the entire process is longer than in the case of brachytherapy, which also discourages older patients. It also happens that older patients, in particular, who initially qualified for proton therapy, were ultimately treated with brachytherapy because their health condition did not allow for proton therapy, e.g., due to the inability to focus the eyes or adopt a forced body position. Interest is also aroused by a very high proportion of patients with retinal detachment among those treated with I-125 brachytherapy—56 patients (55.45%) (Table 1). This is most likely related to the large size of the tumors that qualified for this treatment, as larger tumor size predisposes to retinal detachment [51].

Moving on to the baseline characteristics of the tumors, it is worth noting that groups of tumors treated with different methods differ significantly in all parameters concerning their baseline characteristics (Table 2). This is understandable because the selection of the treatment method is based mainly on the characteristics of the tumor, and the study is not randomized. Interestingly, proton therapy was mainly used to treat small tumors, although this method can also be used for larger tumors. The answer to why this is so lies in the comparison of tumors treated with different methods in terms of their location. Due to the high cost of proton therapy, qualification for it is reserved primarily for patients with a tumor close to the macula and optic disc, so those who have the highest risk of a significant deterioration of visual acuity due to irradiation consequences on these critical structures. At the same time, because of their location, tumors in the posterior pole usually cause symptoms such as decreased vision earlier than tumors located anterior to the equator, so they are also detected earlier, which results in their smaller size at the time of diagnosis. The comparison of numbers of tumors treated with Ru-106 brachytherapy and I-125 brachytherapy in locations close to the optic disc and macula illustrates the greater number of patients treated with Ru-106 brachytherapy, which is also related to their earlier detection, smaller size and, thanks to this, the possibility of treating them with Ru-106 brachytherapy.

### 4.3. Treatment Outcomes

The mean follow-up time was 88.63 months (median 89, range: 20–127). This result is longer (often several times) than in the vast majority of studies devoted to this topic [21,22,23,24,25,27,28,31,35,38,39,40,41,42,43]. It is worth noting that, only in the case of patients who required enucleation, the observation period could be shorter than 5 years. All other patients were followed for a minimum of 5 years from the first day of primary treatment. This allowed for the recording of complications occurring at a later time after treatment and the assessment of visual acuity in the long term.

For the entire group of patients, local tumor control was achieved in 272 patients (90.67%) during the follow-up period. The local tumor control rate was 88.51% in Ru-106 CCB brachytherapy, 89.47% in Ru-106 COB brachytherapy, 91.18% in I-125 brachytherapy and 93.15% in proton therapy. There was no statistically significant difference between the different treatment methods. These results are similar to those of other researchers [3,13,28,30,39]. We would like to emphasize that the assessment of treatment effectiveness was performed only peripherally, as the study was focused on assessing ocular complications. The initial exclusion of patients with other ocular comorbidities and those with a follow-up period of less than 5 years means that the obtained results should be treated with caution because they may differ slightly when considering all treated patients. The decision to enucleate was influenced by the complications identified, their severity and the associated symptoms, e.g., pain, itching of the eye, and the deterioration of visual acuity, but also the patient’s feelings about the appearance of the eye after treatment. The primary cause of enucleation in this case was secondary glaucoma, which was diagnosed in 11 of 14 patients undergoing treatment and was usually associated with severe eye pain. It is worth noting that all of the complications described also led to a loss of vision in the treated eye, with as many as 11 of 14 patients experiencing a loss of light perception prior to eyeball removal. The lack of vision in the treated eye and its unsightly appearance in the patient’s opinion additionally contributed to the patients’ decision to consent to enucleation. In summary, the decision to remove the eyeball due to complications was most often based on several factors. Distant metastasis, noted in only 13 patients (4.33%), should also be approached with great caution because patients who, among other reasons due to metastasis, failed to survive for at least 5 years after initial treatment were excluded from the study. Moreover, it is a collective approach, taking into account all treatment methods together. At the same time, previous studies also show very large variations in the frequency of metastases [3,24,31,35].

### 4.4. Visual Acuity

The deterioration of visual acuity after radiotherapy for uveal melanoma is a huge problem. Among all patients included in our study, BCVA better than or equal to 0.5 was present in 63 patients (21%) at last follow-up. BCVA of 0.1 or worse at the last follow-up characterized 203 patients (67.67%). In both cases, these results were weaker compared to the study by Pors et al., in which it was 47% and 31% respectively. However, the cited study included patients with much better baseline visual acuity, treated only with Ru-106 brachytherapy and with a shorter follow-up period [30]. In the study by Marinkovic et al., which was devoted to patients treated with proton therapy for large tumors and/or their juxtapapillary location, visual acuity at 5 years after treatment was <0.5 in 89.2% of patients and <0.1 in 78.9% of patients [24]. In the study by Wilson and Hungerford, 55% of the patients treated with Ru-106 brachytherapy, 39% of the patients treated with I-125 brachytherapy and 15% of the patients treated with proton therapy had a visual acuity of 6/12 or better at last examination [18]. Such a significant deterioration in eye function is primarily the result of radiation complications, but it is worth remembering that the impact of the tumor itself, causing, e.g., retinal detachment, also contributes to this [51]. While conducting our study, we devoted a lot of attention and analyses to the deterioration of visual acuity and risk factors for its occurrence after brachytherapy and proton therapy of uveal melanoma. Due to the large number of results and conclusions, we decided to devote a separate paper to this issue, which we plan to publish soon. The above paragraph and Table 4 are therefore only a general outline of the results.

### 4.5. Complications and Factors Influencing Their Occurrence

Moving on to the discussion of the most important part of our study, i.e., the factors influencing the occurrence of radiation ocular complication, we would like to emphasize several things. Please note the often numerous differences between our study and the studies whose results we cite in terms of patient inclusion and exclusion criteria, definitions of complications, treatment method, and different lengths of follow-up periods. Please pay attention that below we are comparing the incidence of complications for our entire study group of patients treated with various methods with studies that usually used a single method. For a more comprehensive comparison of complication rates, we encourage you to refer to Table 5, which presents the distribution of complications by treatment method. We also want to draw your attention to the fact that, in the case of multivariable analyses for qualitative variables, it was necessary to select a reference group. If there were only two variants within a variable (e.g., sex—male or female), we simply compared them. For variables with multiple variants, we selected the statistically optimal variant as the reference point. However, the need to select the variant of the variable to which we compared the remaining variants meant that some of them were not compared. Therefore, it cannot be ruled out that additional statistically significant relationships may exist that were not demonstrated in our analysis.

#### 4.5.1. Cataract

Cataract was diagnosed in 151 patients (84.36%) among 179 analyzed patients during follow-up and was the most frequently reported complication. This frequency is higher than in other studies but at the same time our study is characterized by a longer observation period [26,28,30,33,41]. Only older age was found to be statistically significant for a higher risk of cataract development. This parameter also turned out to be a risk factor for the development of cataract in the studies by Espensen et al. and Seibel et al. [41,52]. Greater largest base dimension and near-maximum dose delivered to the lens are remaining risk factors for this complication identified by Espensen et al. [41]. Puusaari et al. showed increasing tumor height to be a risk factor for cataract development [28]. Other researchers have pointed out the key role of the larger tumor size, especially its height and anterior location [53]. Tarmann et al. reported anterior tumor location, and also an elevated irradiation dose (90 Gy) to the tumor apex, as risk factors for cataract development [54]. It is worth noting that, in our study, the association with a higher risk of cataract among patients with a baseline BCVA in the range < 0.1, no light perception > compared with patients with a baseline BCVA ≥ 0.5 reached borderline statistical significance. We have not found poorer baseline BCVA as a risk factor for cataract in other studies. Taking into account the age of the studied patients and the time of cataract appearance in the population of patients not treated with radiotherapy, it seems reasonable to suspect that some of the cases of this diagnosis are simply due to the age of the patients. The positive aspect is that, although cataract is the most common complication in our study group, it can be easily treated surgically.

#### 4.5.2. Maculopathy

Maculopathy turned out to be the second most common complication in our study. A total of 158 patients (52.67%) developed maculopathy during follow-up. This is almost identical to the result of the study by Puusaari et al., in which the 5-year cumulative incidence of maculopathy was 52% [28]. Studies by Espensen et al. and Summanen et al. on patients treated with ruthenium brachytherapy showed a maculopathy rate of 29% and 24%, respectively [41,53]. In a systematic review examining brachytherapy and proton therapy, Tseng et al. reported a maculopathy incidence ranging from 8% to 56% [26]. In multivariate analysis, younger age turned out to be a risk factor for the development of maculopathy, similar to Chang et al.’s study [27]. Tumor location also proved to be statistically significant, because patients with tumors involving the macula and/or optic disc were much more likely to develop maculopathy than those with tumors located at the equator or anterior to the equator, which is consistent with the results of other researchers [53,54]. Tarmann et al. also showed a mushroom tumor shape and pre-existing retinal detachment as risk factors for this complication [54]. In our study, the shape of the tumor did not prove to be a risk factor, and we did not analyze the effect of pre-existing retinal detachment on this topic. In contrast to Puusaari et al.’s results, in our study female sex turned out to be a risk factor for maculopathy [28]. Our results showed that moderately pigmented tumors compared with heavy-pigmented tumors were associated with an increased risk of maculopathy. Interestingly, the presence of systemic hypertension was associated with a lower risk of developing this complication. Unfortunately, we did not find any results regarding the influence of these factors in other publications. Moreover, I-125 brachytherapy was found to be associated with a higher risk of maculopathy than Ru-106 COB brachytherapy.

#### 4.5.3. Retinopathy

The third most common complication was retinopathy, which was diagnosed in 140 patients (46.67%) during follow-up. In the studies by Hasegawa et al., Seibel et al., and among patients treated with I-125 brachytherapy in Guleser et al.’s study, the incidence was 54.7%, 68.1% and 39.3% respectively [25,35,55]. Taking into account patients treated with I-125, Ru-106 brachytherapy and proton therapy in Zemba et al.’s review paper, the incidence of retinopathy falls within a wide range, 10–68.1% [17]. Seibel et al. pointed to the central location of tumor and greater tumor thickness among midperipheral and peripheral tumors as independent risk factors for radiation retinopathy [55]. The results of our study also showed the key role of location, because tumors located anterior to the equator were associated with a substantially lower risk of this complication in comparison to tumors involving the macula and/or optic disc. In our study, tumor thickness did not prove to be a risk factor for retinopathy, but unlike Seibel et al. we did not perform separate analyses for patients with tumors in different locations. Younger age was found to be statistically significant for a higher risk of retinopathy development, similar to the study conducted by Chang et al. [27]. Younger age, distance to optic nerve less than 6 mm and increasing the maximum dose to the fovea were found to be risk factors for retinopathy in Hasegawa et al.’s study [25]. In multivariable analysis, Ru-106 COB brachytherapy was associated with an evidently reduced risk of retinopathy compared to I-125 brachytherapy, as it turned out for maculopathy. Additionally, we would like to mention that, interestingly, the presence of systemic hypertension was found to be of borderline statistical significance as a factor preventing the development of retinopathy, and light-pigmented or amelanotic tumor was found to be of borderline statistical significance as a factor favoring development of this complication compared to heavy-pigmented tumor. We did not find similar results in the studies we assessed.

#### 4.5.4. Optic Neuropathy

A total of 88 patients (29.33%) developed optic neuropathy during follow-up. In the studies by Hasegawa et al., Seibel et al. and Pagliara et al., the incidence was 20.4%, 41.0% and 5.4% respectively [25,55,56]. Risk factors for optic neuropathy in Hasegawa et al.’s study were White race (vs. Others), distance to optic nerve of less than 6 mm as well as increasing the integral radiation dose in the eye, while in Seibal et al.’s study these were a short distance to the optic disc and the dose applied to the optic disc [25,55]. In our study, tumors located within the equator of the eyeball and anterior to it were associated with a decrease in the risk of optic neuropathy compared to locations involving the macula and/or optic disc. An increase in the tumor’s largest base diameter was associated with a higher risk of developing this complication. The finding of these two risk factors is consistent with Tarmann et al.’s results [54]. Our results also indicate, in the case of optic neuropathy, that younger age and the use of I-125 brachytherapy compared to the use of Ru-106 brachytherapy with CCB and COB plaques as risk factors for this complication.

#### 4.5.5. Secondary Glaucoma

Different researchers have different approaches to defining and assessing glaucoma as a complication of radiotherapy [17,26]. Some of them assess only neovascular glaucoma as a complication, while others treat it more generally and assess secondary glaucoma as a complication [20,25,30,54]. We chose the second, more general solution. A total of 83 patients (27.7%) developed secondary glaucoma during follow-up. It was most common among patients treated with I-125 brachytherapy (55 patients, 53.92%) and least common among patients treated with Ru-106 COB brachytherapy (2 patients, 5.26%). In the studies by Tarmann et al., Pors et al., Puusaari et al. and Schönfeld et al., the incidence of this complication was 10.5%, 15.2%, 60% and 23.5% respectively [28,30,54,57]. It is worth noting that the studies by Tarmann et al. and Pors et al. concerned the use of Ru-106 brachytherapy, while Puusaari et al. concerned the use of I-125 brachytherapy and Schönfeld et al. concerned proton therapy [28,30,54,57]. Tarmann et al. show a mushroom-like tumor shape and a higher tumor distance to the optic disc as risk factors while Pors et al. pointed out that glaucoma incidence was correlated with tumor ciliary body involvement [30,54]. A greater radiation dose to the opposite retina, tumor height and IOP at diagnosis were associated with secondary glaucoma in Puusaari et al.’s studies [28,58]. Our study also found that greater tumor thickness was associated with an increased risk of secondary glaucoma. Furthermore, a higher incidence of glaucoma was associated with baseline BCVA weaker than 0.5 and the use of I-125 brachytherapy compared to Ru-106 CCB brachytherapy. It is worth paying attention to the fact that, in our study, similarly to the studies cited above, the incidence of glaucoma among patients treated with Ru-106 brachytherapy was several times lower compared to I-125 brachytherapy. To our great surprise, the location of the tumor in the left eye compared to the right eye was also associated with a higher risk of developing secondary glaucoma in multivariate analysis. Unfortunately, we have not found similar results in the available publications regarding the impact of poor baseline visual acuity and tumor location in the left eye on the development of secondary glaucoma. Regarding the higher risk associated with poorer baseline visual acuity, a clue could be the higher risk of developing glaucoma in patients with myopia and hyperopia but we have not analyzed it in detail yet [59,60]. Unfortunately, we currently do not know how to explain the higher risk of developing secondary glaucoma when the left eye is affected.

#### 4.5.6. Vitreous Hemorrhage

Of the 295 patients analyzed, 56 (19%) were diagnosed with vitreous hemorrhage during the follow-up. In Hasegawa et al.’s study, it occurred in 6.7% of patients [25]. In Tarmann et al. and Summanen et al.’s studies, it was 17.5% and 15% respectively [53,54]. Risk factors for vitreous hemorrhage that showed statistical significance in multivariable analyses performed by other investigators include: being of White race, the integral radiation dose, tumor thickness more than 5 mm and a mushroom-shaped tumor [25,53,54]. Our results regarding tumor parameters were largely consistent with those obtained in the studies mentioned above. In our study, the following were associated with a higher risk of vitreous hemorrhage: greater tumor thickness, a mushroom-shaped tumor compared to a domed shape, and the tumor location including the macula and/or optic disc compared to all other locations.

#### 4.5.7. Hyphema

While reviewing the literature, we did not find hyphema as a complication following radiotherapy for uveal melanoma. At the same time, since we decided to present all complications that occurred in the group of patients we studied, and the relationship between the occurrence of hyphema and radiotherapy was clear, we present it as a post-radiation complication of the treatment analogously to vitreous hemorrhage. During the follow-up period, hyphema was diagnosed in 25 patients (8.3%) and was most common among patients treated with I-125 brachytherapy (17 patients, 16.67%) and least common among patients treated with Ru-106 CCB brachytherapy (1 patient, 1.15%). Unfortunately, due to the insufficient number of identified cases, we refrained from performing univariable and multivariable analyses of potential risk factors for this complication because their credibility would be low.

#### 4.5.8. Retinal Detachment

Of the 222 patients analyzed, 17 (7.7%) were diagnosed with retinal detachment during the follow-up. In the studies by Espensen et al. and Puusaari et al., the incidence of this complication was respectively 36% and 25% [23,28]. In the Tarmann et al. study, retinal detachment occurred in 18.9% of patients but they include both those who developed a retinal detachment or where retinal detachment increased [54]. Zemba et al., in their systematic review, report the incidence of this complication from 7.3% to 38%, varying for different treatment methods [17]. Differences in the criteria for assessing this complication between different studies make their comparison difficult. Unfortunately, due to the insufficient number of identified cases, we refrained from performing univariable and multivariable analyses of potential risk factors for this complication because their credibility would be low. It is worth noting that exudative retinal detachment diagnosed before radiotherapy of the tumor often resolves within a few or a dozen months after treatment [17].

#### 4.5.9. Keratopathy

There are not many studies devoted to the occurrence of radiation-induced complications of the ocular surface after radiotherapy for uveal melanoma, except those devoted to iris melanoma. This may be due to the fact that they usually do not have as strong a negative impact on visual acuity as, for example, optic neuropathy or maculopathy, or perhaps less importance is attached to examining patients for them or they simply occur less frequently. In our study only seven patients (2.3%) developed keratopathy during follow-up. Keratopathy was most common among patients treated with I-125 brachytherapy (6 patients, 5.88%) and least common among patients treated with Ru-106 brachytherapy with CCB and COB plaques, in which no patient was diagnosed with this complication. In Convay et al.’s study, the incidence of keratopathy or dry eye was 23.8% [61]. We have included corneal abnormalities resulting from radiotherapy, e.g., corneal haze, in the broad concept of keratopathy, but some researchers use the narrower term to assess corneal complications, which is keratitis. The incidence of it in Quivey et al.’s study was 3.8% [62]. Unfortunately, due to the small number of identified cases, we refrained from performing univariable and multivariable analyses of potential risk factors for this complication because their credibility would be low.

#### 4.5.10. Dry Eye

As in the case of keratopathy, there are not many studies devoted to dry eye after radiotherapy for uveal melanoma. In our study only four patients (1.3%) developed dry eye during follow-up. It was most common among patients treated with proton therapy (2 patients, 2.74%) and least common among patients treated with Ru-106 brachytherapy with CCB and COB plaques, in which no patient was diagnosed with this complication. In Thariat et al.’s study, a five-year incidence of dry eye syndrome and severe dry eye syndrome was 23.0% and 10.9% respectively [63]. Quivey et al. reported an 8% incidence of dry eye after I-125 brachytherapy [62]. Ocular surface complications such as dry eye are considered more common with proton therapy than with brachytherapy [17]. We suspect that the significantly lower incidence of dry eye syndrome among our patients than in the cited studies may be due to its underdiagnosis, as we did not routinely perform tests for it during the follow-up, such as the Schirmer test and tear break-up time (TBUT) test. Therefore, its true incidence may be higher. Unfortunately, in this case too, due to the small number of identified cases, we refrained from performing univariable and multivariable analyses of potential risk factors for this complication because their credibility would be low.

To summarize, the most important factors which influenced the development of the four complications were the patient’s age (younger age increased the risk of maculopathy, retinopathy, and optic neuropathy, and older age increased the risk of cataract), the location of the tumor (maculopathy, retinopathy, optic neuropathy and vitreous hemorrhage), and the treatment method used (maculopathy, retinopathy, optic neuropathy and secondary glaucoma). Next in line was tumor thickness, the increase in which was associated with a higher risk of two complications (secondary glaucoma, vitreous hemorrhage). The remaining factors in the multivariable analysis influenced the occurrence of one complication, except for the presence of diabetes at baseline, the role of which was not statistically significant in the development of any complication. It is worth noting that the treatment method used and the thickness of the tumor are closely related, because the treatment method is largely selected based on the thickness of the tumor [8,17,20,26,29].

#### 4.5.11. Summary of Number of Complications

The overall summary of the number of complications per patient shows a very large variation in this regard. This number varied greatly from 36 patients (12%) in whom no complications of radiotherapy were observed throughout the entire follow-up period to as many as eight complications in two patients (0.67%). Hasegawa et al. presented results in which as many as 41.8% of patients had no complications, while their maximum number was four and was found in only 0.9% of patients. However, it should be noted that, compared to our study, the Hasegawa et al. study included only patients treated with iodine, and the median follow-up period was almost three times shorter than in our study (33.6 months vs. 89 months) [25].

### 4.6. Treatment of Complications of Radiotherapy

Over time, we have gained access to newer and more advanced methods of treating radiation-related complications [64,65]. Unfortunately, we still cannot cope effectively with all of them. Cataract surgery (94 patients) and anti-VEGF injections (91 patients) were the most common treatments for radiation complications among the patients we analyzed. This corresponds to the most common complications in our study, cataract, maculopathy and retinopathy, and also those frequently reported by many other researchers [17,20,26,28,30]. All patients had an opportunity to benefit from treatment for complications, whenever medically possible. In this study, we did not compare the outcomes of patients who received treatment for complications and those who did not. However, we are considering conducting such an analysis in the future, with particular emphasis on the impact on visual acuity.

### 4.7. Treatment of Failures of Radiotherapy

Analysis of the effectiveness of retreatment in the absence of efficacy of primary treatment was not the subject of this study. However, it is worth emphasizing that subsequent radiotherapy treatment due to failure of the primary treatment is associated with an additional dose of radiation and, therefore, it also contributes to the occurrence of radiation-related complications. Additional TTT or other methods used also have an impact on the condition of the eye. We decided not to exclude from the analysis patients who required repeated treatment, even though to some extent they most likely worsen the results in terms of the number of post-radiation complications and visual acuity, because in fact this is the result of imperfections in the initial treatment. It is worth remembering this when analyzing the results of this and other studies. Unfortunately, except for Tarmann et al.’s study, none of the studies we found on radiation complications included detailed information on the outcomes of patients who underwent repeated radiotherapy due to failure of the primary treatment, and most often there is no mention of such patients [54].

### 4.8. Strengths and Limitations

The strengths of our study include a relatively large number of analyzed patients, despite the use of very restrictive inclusion and exclusion criteria. Their use was intended to obtain the most reliable results as possible into the actual impact of the analyzed factors. Another advantage is the long follow-up period of 88.63 months on average (median 89 months), which is often several times longer than in many studies on ocular radiation complications we found and cited above. Thanks to this, we were able to record a greater number of complications that appeared later. Another advantage of the study is its detailed nature. We recorded the occurrence of all diagnosed complications, not just selected ones. We analyzed the influence of as many as 12 factors that could influence the occurrence of the six most common complications. Analyses of the influence of factors on the occurrence of the four remaining rarest complications were not performed, only because of their small number, which prevented a reliable analysis. Another advantage was the use of the radiotherapy method as a risk factor for ocular complications, which is rarely practiced. A similar method was used by Shields et al. and Aziz et al. but to assess the effect on visual acuity [20,43]. Also unique is the inclusion of as many as three treatment methods in one study. Taking into account the division of brachytherapy into two types depending on the applicator used, which was aimed at finding possible differences between them, it can be treated as an analysis of four therapy ways.

Our study also has weaknesses that we would like to point out. This is a retrospective study and therefore it has imperfections typical of this type of research. One of them is the lack of some data. Patients sometimes missed their scheduled follow-up appointments for various reasons and often showed up for the examination with a delay of several months or more. This is important when assessing the time to onset of complications, as we can assume that their presence would have been detected earlier if all planned follow-up visits had taken place. This leads to a longer average time from treatment to the onset of complications. Generally, however, in all studies of this type, we can assume that complications appear sooner than the analysis suggests, because of the delay in their detection due to the intervals between follow-up visits. It is important to note that over the years, patients were examined and treated by different doctors, nurses, and support staff. The medical equipment used has also partially changed. Despite the consistency of procedures, assessment criteria, and decision-making recommendations, it must be assumed that they were not entirely identical for all patients. This seems inevitable for retrospective studies in real-world hospital settings, especially if part of the decision, e.g., regarding the choice of therapy or treatment of complications, rests with the patient. Differences also exist in the treatment of complications. Patients at different times opted for the proposed procedures, such as cataract surgery or anti-VEGF injections. Moreover, patients typically had the option of having their complications treated at our center or closer to their home, often hundreds of kilometers away. Therefore, many patients opted for treatment of complications in hospitals closer to their home. On the one hand, such non-identical procedures may have some impact on the study results, but on the other hand, it greatly facilitates the treatment convenience for patients, and their well-being is paramount.

### 4.9. Clinical and Research Implications

Many researchers are working on improving radiotherapy for uveal melanoma in order to reduce the number of radiation-related complications and maintain better visual acuity [66]. At the same time, some studies show that a solution that improves visual outcomes may risk reducing local control and increasing mortality [67]. Therefore, it is a kind of balancing act in search of the golden mean that simultaneously combines improved results in terms of complications and the preservation of vision, as well as local control.

Among the factors with the greatest influence on the development of complications in our study, it is worth noting that the patient’s age and tumor location are non-modifiable factors. The treatment method used and the thickness of the tumor are closely related, because as mentioned above the treatment method is largely selected based on the thickness of the tumor [8,17,20,26,29]. The treatment method is a modifiable factor, and the tumor thickness status, seemingly unmodifiable, may not be obvious. New research on systemic treatment also offers hope for reducing tumor thickness, which is thus becoming a modifiable factor. This may enable the use of more sparing methods of radiotherapy, increasing the chances of preserving useful vision [68,69]. The radiation dose to structures critical for vision, such as the macula and optic disc, is also related to the location and size of the tumor and the treatment method used. Higher doses of radiation absorbed by these structures are associated with a greater risk of complications and decreased visual acuity [22]. Among others, sensitizing cancer cells to radiation seems to be a promising approach, which would make it possible to limit the necessary radiation dose delivered to the tumor and thus also to reduce the dose for the surrounding healthy tissues [70,71]. However, we must remember that due to the existing risk to the patient’s life, we should always approach new vision-saving methods with great caution.

In our opinion, one of the key elements to achieving the goal of reducing the number of complications and maintaining good visual acuity after radiotherapy for uveal melanoma is the precise identification and very good understanding of the factors that have an adverse effect on eye function. Knowing them allows us to optimize the direction of further research. We believe that the results we present in this article successfully fulfill this role. We are also working on a paper dedicated to the assessment of demographic, clinical and physical risk factors for visual acuity deterioration, which will complement the current article and contribute to defining key factors associated with the deterioration of eye function after radiotherapy.

## 5. Conclusions

Our study demonstrated an association between demographic, clinical, and physical factors and the occurrence of ocular complications after radiotherapy for uveal melanoma. The most important factors were the patient’s age, location of the tumor, treatment method used and to a lesser extent the tumor thickness. The remaining analyzed factors played a lesser role. We believe that the results of our study may contribute to the improvement in existing, and the development of new, therapeutic methods that reduce the number of complications and preserve visual acuity while maintaining or improving local tumor control after radiotherapy for uveal melanoma.

## Figures and Tables

**Table 1:** Baseline patients characteristics (N = 300).

		**Treatment Method**				
Characteristics	Total (N = 300)	Ru-106 CCB (n = 87)	Ru-106 COB (n = 38)	I-125 (n = 102)	PT (n = 73)	*p* Value
Age (years)	58.5 ± 12.94 [60 (22–85)]	61.69 ± 11.54 a [61 (32–85)]	64.05 ± 10 a [64.5 (37–83)]	58.24 ± 12.65 ab [58.5 (23–81)]	52.19 ± 13.84 b [56 (22–81)]	<0.00001 ^
Sex						0.07862 #
Female	169 (56.33)	48 (55.17)	28 (73.68)	58 (56.86)	35 (47.95)	
Male	131 (43.67)	39 (44.83)	10 (26.32)	44 (43.14)	38 (52.05)	
Systemic hypertension						0.47444 #
No	152 (50.67)	41 (47.13)	16 (42.11)	56 (54.90)	39 (53.42)	
Yes	148 (49.33)	46 (52.87)	22 (57.89)	46 (45.10)	34 (46.58)	
Diabetes mellitus						0.79294 #
No	270 (90.00)	78 (89.66)	33 (86.84)	94 (92.16)	65 (89.04)	
Yes	30 (10.00)	9 (10.34)	5 (13.16)	8 (7.84)	8 (10.96)	
Affected eye						0.29638 #
Right	154 (51.33)	52 (59.77)	17 (44.74)	50 (49.02)	35 (47.95)	
Left	146 (48.67)	35 (40.23)	21 (55.26)	52 (50.98)	38 (52.05)	
Cataract						0.19021 #
No	179 (59.67)	48 (55.17)	20 (52.63)	57 (55.88)	54 (73.97)	
Yes	102 (34.00)	32 (36.78)	15 (39.47)	39 (38.24)	16 (21.92)	
IOL	19 (6.33)	7 (8.05)	3 (7.89)	6 (5.88)	3 (4.11)	
Retinal detachment	78 (26)	10 (11.49)	1 (2.56)	56 (55.45)	11 (15.07)	<0.0001 #
Vitreous hemorrhage	5 (1.67)	0 (0)	0 (0)	5 (4.90)	0 (0)	0.01970 #
Family history of cancer						0.28330 #
No	214 (71.33)	61 (70.11)	32 (84.21)	72 (70.59)	49 (67.12)	
Yes	86 (28.67)	26 (29.89)	6 (15.79)	30 (29.41)	24 (32.88)	
Follow-up duration (months)	88.63 ± 19.31 [89 (20–127)]	89.32 ± 18.84 ab [88 (22–127)]	88.26 ± 13.78 ab [85.5 (59–119)]	83.97 ± 19.76 a [86 (20–126)]	94.52 ± 20.33 b [99 (21–126)]	0.00030 ^

Qualitative variable as n (%); quantitative variables: mean ± SD (median; minimum–maximum); #—Chi-square test; ^—Kruskal–Wallis test. Groups not sharing the same letter (a, b) differ statistically significantly at *p* < 0.05; Dunn’s post hoc test. Definition of abbreviations: Ru-106 CCB = Ruthenium-106 brachytherapy with CCB plaque, Ru-106 COB = Ruthenium-106 brachytherapy with COB plaque, I-125 = Iodine-125 brachytherapy, PT = proton therapy.

**Table 2:** Baseline tumors characteristics (N = 300).

		**Treatment Method**				
Characteristics	Total (N = 300)	Ru-106 CCB (n = 87)	Ru-106 COB (n = 38)	I-125 (n = 102)	PT (n = 73)	*p* Value
Thickness (mm)	4.73 ± 2.92 [3.53 (0.77–13.4)]	3.24 ± 1.04 a [3.1 (1.35–6.7)]	2.44 ± 0.8 b [2.39 (0.77–5.1)]	8.18 ± 2.06 c [7.8 (4.7–13.4)]	2.89 ± 1.44 ab [2.4 (1.3–9.3)]	<0.00001 ^
Tumor largest base diameter (mm)	11.06 ± 3.15 [10.7 (3.17–18.8)]	10.45 ± 2.49 a [10.5 (3.9–17.1)]	8.17 ± 1.85 b [8.5 (3.17–11.3)]	13.91 ± 2.39 c [13.9 (8.6–18.8)]	9.32 ± 2.11 b [8.9 (5.4–14.9)]	<0.00001 ^
AJCC classification						<0.00001 #
T1	134 (44.67)	41 (47.13)	36 (94.74)	1 (0.98)	56 (76.71)	
T2	85 (28.33)	43 (49.43)	2 (5.26)	25 (24.51)	15 (20.55)	
T3	75 (25.00)	3 (3.45)	0 (0)	70 (68.63)	2 (2.74)	
T4	6 (2.00)	0 (0)	0 (0)	6 (5.88)	0 (0)	
COMS classification						<0.00001 #
Small	72 (26.18)	18 (24.00)	16 (55.17)	0 (0)	38 (52.05)	
Medium	166 (60.36)	56 (74.67)	13 (44.83)	62 (63.27)	35 (47.95)	
Large	37 (13.46)	1 (1.33)	0 (0)	36 (36.73)	0 (0)	
Location						<0.00001 #
Macula and/or optic disc	54 (44.67)	14 (16.09)	5 (13.16)	10 (9.80)	25 (34.25)	
Posterior to the equator without the macula and optic disc	133 (28.33)	42 (48.28)	15 (39.47)	38 (37.25)	38 (52.05)	
Equator	46 (25.00)	11 (12.64)	4 (10.53)	21 (20.59)	10 (13.70)	
Anterior to the equator	67 (2.00)	20 (22.99)	14 (36.84)	33 (32.35)	0 (0)	
Shape						<0.00001 #
Flat	8 (2.67)	5 (5.75)	3 (7.89)	0 (0)	0 (0)	
Domed	253 (84.33)	80 (91.95)	35 (92.11)	68 (66.67)	70 (95.89)	
Mushroom-shaped	39 (13.00)	2 (2.30)	0 (0)	34 (33.33)	3 (4.11)	
Pigmentation						000811 #
Heavy	64 (21.33)	25 (28.74)	13 (34.21)	15 (14.71)	11 (15.07)	
Moderate	186 (62.00)	44 (50.57)	22 (57.89)	66 (64.71)	54 (73.97)	
Light or amelanotic	50 (16.67)	18 (20.69)	3 (7.89)	21 (20.59)	8 (10.96)	
Dose received by the tumor apex (Gy)	97.33 ± 10.23 [95.58 (73.06–123.4)]	97.48 ± 10.26 [95.39 (80.5–119.6)]	96.7 ± 9.39 [96.81 (80.4–116.5)]	97.43 ± 10.58 [95.55 (73.06–123.4)]	60 CGE *	099999 ^

Qualitative variable as n (%); quantitative variables: mean ± SD (median; minimum–maximum); #—Chi-square test; ^—Kruskal–Wallis test. Groups not sharing the same letter (a, b, c) differ statistically significantly at *p* < 0.05; Dunn’s post hoc test. * Dose prescribed before treatment without post-treatment analysis; definition of abbreviations: mm = millimeter, AJCC classification = American Joint Committee on Cancer classification (8th edition), COMS classification = Collaborative Ocular Melanoma Study classification, Gy = gray, CGE = Cobalt Gray Equivalent.

**Table 3:** Treatment effectiveness table showing failures (N = 300).

		**Treatment Method**				
Treatment Failures	Total (N = 300)	Ru-106 CCB (n = 87)	Ru-106 COB (n = 38)	I-125 (n = 102)	PT (n = 73)	*p* Value
Tumor size progression at first follow-up						0.20876
No	293 (97.67)	86 (98.85)	37 (97.37)	101 (99.02)	69 (94.52)	
Yes	7 (2.33)	1 (1.15)	1 (2.63)	1 (0.98)	4 (5.48)	
Recurrence						0.09649
No	277 (92.33)	77 (88.51)	34 (89.47)	94 (92.16)	72 (98.63)	
Yes	23 (7.67)	10 (11.49)	4 (10.53)	8 (7.84)	1 (1.37)	
Tumor size progression at first follow-up and/or recurrence						0.77524
No	272 (90.67)	77 (88.51)	34 (89.47)	93 (91.18)	68 (93.15)	
Yes	28 (9.33)	10 (11.49)	4 (10.53)	9 (8.82)	5 (6.85)	

Variables presented as n (%); Chi-square test.

**Table 4:** Visual acuity before treatment and after the observation period (N = 300).

Visual Acuity Level	Baseline BCVA	Final BCVA
1.0	71 (23.67)	15 (5.00)
0.9	19 (6.33)	7 (2.33)
0.8	22 (7.33)	6 (2.00)
0.7	26 (8.67)	20 (6.67)
0.6	13 (4.33)	3 (1.00)
0.5	31 (10.33)	12 (4.00)
0.4	22 (7.33)	11 (3.67)
0.3	19 (6.33)	9 (3.00)
0.2	29 (9.67)	14 (4.67)
0.1	9 (3.00)	8 (2.67)
0.05	10 (3.33)	23 (7.67)
Counting fingers	26 (8.67)	51 (17.00)
Hand motions	2 (0.67)	25 (8.33)
Light perception	1 (0.33)	33 (11.00)
No light perception	0 (0)	63 (21.00) *
Visual acuity group		
≥0.5	182 (60.66)	63 (21)
<0.5 and >0.1	70 (23.33)	34 (11.34)
≤0.1	48 (16)	203 (67.67) *

Variables presented as n (%). * Including 20 enucleated eyes; definition of abbreviations: BCVA = best corrected visual acuity.

**Table 5:** Radiation complications found during the follow-up period (N = 300).

		**Treatment Method**				
Complication	Total (N = 300)	Ru-106 CCB (n = 87)	Ru-106 COB (n = 38)	I-125 (n = 102)	PT (n = 73)	*p* Value
Secondary glaucoma						<0.00001
No	217 (72.33)	81 (93.10)	36 (94.74)	47 (46.08)	53 (72.60)	
Yes	83 (27.67)	6 (6.90)	2 (5.26)	55 (53.92)	20 (27.40)	
Maculopathy						0.00011
No	142 (47.33)	43 (49.43)	28 (73.68)	50 (49.02)	21 (28.77)	
Yes	158 (52.67)	44 (50.57)	10 (26.32)	52 (50.98)	52 (71.23)	
Retinopathy						<0.00001
No	160 (53.33)	48 (55.17)	35 (92.11)	47 (46.08)	30 (41.10)	
Yes	140 (46.67)	39 (44.83)	3 (7.89)	55 (53.92)	43 (58.90)	
Optic neuropathy						<0.00001
No	212 (70.67)	73 (83.91)	37 (97.37)	55 (53.92)	47 (64.38)	
Yes	88 (29.33)	14 (16.09)	1 (2.63)	47 (46.08)	26 (35.62)	
Cataract *						0.07330
No	28 (15.64)	9 (18.75)	2 (10.00)	4 (7.02)	13 (24.07)	
Yes	151 (84.36)	39 (81.25)	18 (90.00)	53 (92.98)	41 (75.93)	
Dry eye						0.37766
No	296 (98.67)	87 (100.00)	38 (100.00)	100 (98.04)	71 (97.26)	
Yes	4 (1.33)	0 (0)	0 (0)	2 (1.96)	2 (2.74)	
Vitreous hemorrhage *						0.00144
No	239 (81.02)	79 (90.80)	35 (92.11)	69 (71.13)	56 (76.71)	
Yes	56 (18.98)	8 (9.20)	3 (7.89)	28 (28.87)	17 (23.29)	
Hyphema						0.00079
No	275 (91.67)	86 (98.85)	37 (97.37)	85 (83.33)	67 (91.78)	
Yes	25 (8.33)	1 (1.15)	1 (2.63)	17 (16.67)	6 (8.22)	
Retinal detachment *						0.02925
No	205 (92.34)	74 (96.10)	36 (97.30)	38 (82.61)	57 (91.94)	
Yes	17 (7.66)	3 (3.90)	1 (2.70)	8 (17.39)	5 (8.06)	
Keratopathy						0.03036
No	293 (97.67)	87 (100.00)	38 (100.00)	96 (94.12)	72 (98.63)	
Yes	7 (2.33)	0 (0)	0 (0)	6 (5.88)	1 (1.37)	

Qualitative variables expressed as n (%); Chi-square test; * patients with this abnormality diagnosed before treatment were excluded from this complication analysis.

**Table 6:** Analysis of the influence of selected factors on the occurrence of complications *—univariable Cox proportional hazards regression analyses.

Part 1										
		Secondary Glaucoma			Vitreous Hemorrhage			Maculopathy		
Factor	Value **	HR	95% CI	*p*-Value	HR	95% CI	*p*-Value	HR	95% CI	*p*-Value
Baseline BCVA	<0.5 and >0.1	2.29	1.37–3.83	0.00159	1.95	1.08–3.52	0.02708	1.16	0.81–1.67	0.42261
Baseline BCVA	≤0.1	3.33	1.98–5.61	0.00001	2.24	1.17–4.29	0.01501	1.09	0.71–1.68	0.67972
Tumor pigmentation	Light or amelanotic	1.73	0.81–3.70	0.15675	1.80	0.78–4.17	0.16979	1.81	1.07–3.05	0.02729
Tumor pigmentation	Moderate	1.82	0.98–3.40	0.05883	1.38	0.69–2.78	0.36275	1.74	1.14–2.65	0.01028
Diabetes mellitus	Yes	0.99	0.48–2.05	0.97516	0.84	0.34–2.11	0.71455	0.60	0.32–1.10	0.09902
Tumor largest base diameter (mm)		1.23	1.14–1.32	<0.00001	1.05	0.97–1.14	0.24317	1.03	0.98–1.08	0.18656
Tumor thickness (mm)		1.35	1.27–1.44	<0.00001	1.20	1.11–1.30	0.00001	1.00	0.94–1.05	0.89421
Tumor shape	Domed	0.28	0.17–0.45	<0.00001	0.26	0.15–0.45	<0.00001	1.30	0.80–2.12	0.29129
Tumor shape	Flat	0.16	0.02–1.16	0.06979	0	0–Inf	0.99521	0.44	0.10–1.91	0.27635
Tumor location	Anterior to the equator	1.59	0.83–3.07	0.16494	0.41	0.19–0.9	0.02579	0.40	0.24–0.67	0.00048
Tumor location	Posterior to the equator without the macula and optic disc	0.92	0.49–1.70	0.78119	0.44	0.23–0.81	0.00871	0.88	0.60–1.28	0.50480
Tumor location	Equator	1.67	0.83–3.34	0.14997	0.65	0.31–1.39	0.26935	0.61	0.36–1.03	0.06478
Systemic hypertension	Yes	0.64	0.41–0.99	0.04346	0.78	0.46–1.30	0.34151	0.63	0.46–0.85	0.00301
Affected eye	Left	1.67	1.08–2.57	0.02115	1.28	0.76–2.13	0.35248	1.07	0.79–1.45	0.65285
Sex	Female	0.62	0.40–0.95	0.02968	0.66	0.40–1.11	0.11762	1.51	1.10–2.07	0.01052
Treatment method	PT	0.39	0.23–0.66	0.00039	0.71	0.39–1.3	0.26804	1.51	1.03–2.23	0.03439
Treatment method	Ru-106 CCB	0.09	0.04–0.21	<0.00001	0.27	0.12–0.59	0.00114	0.89	0.60–1.33	0.58116
Treatment method	Ru-106 COB	0.07	0.02–0.28	0.00020	0.23	0.07–0.75	0.01497	0.39	0.20–0.76	0.00609
Age (years)		0.97	0.96–0.99	0.00047	0.97	0.95–0.99	0.00089	0.98	0.97–0.99	0.00418
**Part 2**										
		**Optic Neuropathy**			**Retinopathy**			**Cataract**		
**Factor**	Value **	**HR**	**95% CI**	***p*** **-Value**	**HR**	**95% CI**	***p*** **-Value**	**HR**	**95% CI**	***p*** **-Value**
Baseline BCVA	<0.5 and >0.1	1.57	0.97–2.55	0.06716	1.51	1.03–2.20	0.03322	1.57	1.08–2.30	0.01855
Baseline BCVA	≤0.1	2.09	1.25–3.49	0.00493	1.64	1.06–2.53	0.02625	2.14	1.37–3.33	0.00077
Tumor pigmentation	Light or amelanotic	2.02	0.96–4.28	0.06571	2.81	1.6–4.94	0.00032	1.11	0.65–1.87	0.70644
Tumor pigmentation	Moderate	2.13	1.15–3.94	0.01644	2.01	1.24–3.26	0.00442	1.31	0.87–1.99	0.19681
Diabetes mellitus	Yes	0.77	0.36–1.67	0.51267	0.82	0.45–1.47	0.50047	1.41	0.81–2.46	0.21897
Tumor largest base diameter (mm)		1.17	1.10–1.25	<0.00001	1.06	1.01–1.11	0.02467	1.11	1.05–1.17	0.00037
Tumor thickness (mm)		1.15	1.08–1.23	0.00001	1.07	1.02–1.13	0.00896	1.14	1.08–1.21	<0.00001
Tumor shape	Domed	0.62	0.36–1.06	0.08022	0.52	0.34–0.80	0.00322	0.62	0.40–0.97	0.03710
Tumor shape	Flat	0.24	0.03–1.84	0.17072	0.37	0.11–1.22	0.10334	0.23	0.05–0.99	0.04784
Tumor location	Anterior to the equator	0.37	0.18–0.73	0.00402	0.28	0.15–0.52	0.00006	1.31	0.78–2.2	0.31556
Tumor location	Posterior to the equator without the macula and optic disc	0.60	0.36–0.98	0.04246	0.85	0.57–1.27	0.42222	1.22	0.80–1.86	0.35609
Tumor location	Equator	0.75	0.40–1.39	0.35726	0.95	0.58–1.57	0.85501	0.78	0.45–1.36	0.38539
Systemic hypertension	Yes	0.85	0.57–1.28	0.44538	0.56	0.40–0.78	0.00060	0.87	0.63–1.20	0.38813
Affected eye	Left	1.53	1.01–2.3	0.04382	1.18	0.85–1.63	0.31764	0.98	0.71–1.34	0.87760
Sex	Female	0.91	0.60–1.37	0.64578	1.00	0.72–1.39	0.99783	1.34	0.97–1.84	0.07207
Treatment method	PT	0.68	0.42–1.09	0.11095	0.93	0.62–1.40	0.73795	0.47	0.31–0.71	0.00037
Treatment method	Ru-106 CCB	0.26	0.14–0.48	0.00001	0.62	0.41–0.94	0.02375	0.48	0.31–0.73	0.00060
Treatment method	Ru-106 COB	0.04	0.01–0.30	0.00156	0.09	0.03–0.29	0.00006	0.66	0.38–1.13	0.12534
Age (years)		0.98	0.96–0.99	0.00408	0.98	0.97–0.99	0.00069	1.02	1.01–1.03	0.00851

* Due to the insufficient number of cases identified, analyses for hyphema, retinal detachment, keratopathy and dry eye were abandoned. ** The following reference values were selected for qualitative variables: baseline BCVA—≥0.5, tumor pigmentation—heavy, diabetes mellitus—no, tumor shape—mushroom-shaped, tumor location—macula and/or optic disc, systemic hypertension—no, affected eye—right, sex—male, treatment method—I-125. Definition of abbreviations: HR = hazard ratio, CI = confidence interval.

**Table 7:** Results of multivariable analysis of radiation complication * risk factors that proved statistically significant in the univariable analysis–multivariable Cox proportional hazards regression analyses.

Complication, Variable and Value **	HR	95% CI	*p* Value
**Secondary glaucoma**			
Sex-Female	0.94	0.59–1.47	0.77292
Age (years)	0.99	0.97–1.01	0.15277
Treatment method—PT	1.71	0.67–4.37	0.26182
**Treatment method—Ru-106 CCB**	**0.34**	**0.11–1.00**	**0.04928**
Treatment method—Ru-106 COB	0.30	0.06–1.58	0.15559
**Affected eye—Left**	**1.77**	**1.12–2.80**	**0.01462**
Systemic hypertension—Yes	0.93	0.56–1.53	0.76725
**Tumor thickness (mm)**	**1.28**	**1.10–1.47**	**0.00092**
Tumor largest base diameter (mm)	0.98	0.87–1.11	0.77298
Tumor shape—Domed	1.02	0.53–1.96	0.95029
Tumor shape—Flat	3.25	0.33–32.08	0.31326
**Baseline BCVA—<0.5 and >0.1**	**2.14**	**1.20–3.81**	**0.00984**
**Baseline BCVA—≤0.1**	**2.43**	**1.34–4.42**	**0.00363**
**Vitreous hemorrhage**			
Age (years)	0.98	0.96–1.00	0.10412
Treatment method—PT	2.96	0.90–9.71	0.07345
Treatment method—Ru-106 CCB	1.57	0.47–5.23	0.46209
Treatment method—Ru-106 COB	2.09	0.41–10.53	0.37294
**Tumor thickness (mm)**	**1.28**	**1.07–1.53**	**0.00779**
**Tumor location—Anterior to the equator** **(vs. the macula and/or optic disc)**	**0.26**	**0.10–0.68**	**0.00627**
**Tumor location—Posterior to the equator ** **without the macula and optic disc**	**0.36**	**0.18–0.72**	**0.00416**
**Tumor location—Equator**	**0.42**	**0.17–1.00**	**0.04984**
**Tumor shape—Domed**	**0.40**	**0.19–0.82**	**0.01254**
Tumor shape—Flat	0	0–Inf	0.99578
Baseline BCVA—<0.5 and >0.1	1.62	0.83–3.19	0.15882
Baseline BCVA—≤0.1	1.42	0.63–3.19	0.39715
**Maculopathy**			
**Sex—Female**	**2.02**	**1.45–2.83**	**0.00004**
**Age (years)**	**0.99**	**0.97–1.00**	**0.04875**
Treatment method—PT	1.15	0.75–1.76	0.52173
Treatment method—Ru-106 CCB	0.93	0.62–1.41	0.73914
**Treatment method—Ru-106 COB**	**0.37**	**0.18–0.74**	**0.00489**
**Systemic hypertension—Yes**	**0.65**	**0.46–0.93**	**0.01720**
**Tumor location—Anterior to the equator**	**0.43**	**0.24–0.77**	**0.00469**
Tumor location—Posterior to the equator without the macula and optic disc	0.90	0.60–1.36	0.61432
**Tumor location—Equator**	**0.45**	**0.25–0.8**	**0.00643**
Tumor pigmentation—Light or amelanotic (vs. heavy)	1.36	0.76–2.45	0.29776
**Tumor pigmentation—Moderate**	**1.68**	**1.05–2.67**	**0.02903**
**Optic neuropathy**			
**Age (years)**	**0.98**	**0.96–1.00**	**0.01466**
Treatment method—PT	0.65	0.26–1.62	0.35671
**Treatment method—Ru-106 CCB**	**0.31**	**0.13–0.74**	**0.00783**
**Treatment method—Ru-106 COB**	**0.07**	**0.01–0.62**	**0.01625**
Affected eye—Left	1.38	0.89–2.12	0.14662
Tumor thickness (mm)	0.95	0.82–1.11	0.52292
**Tumor largest base diameter (mm)**	**1.14**	**1.02–1.27**	**0.01706**
**Tumor location—Anterior to the equator**	**0.34**	**0.15–0.77**	**0.00919**
Tumor location—Posterior to the equator without the macula and optic disc	0.60	0.34–1.06	0.07772
**Tumor location—Equator**	**0.45**	**0.22–0.94**	**0.03299**
Tumor pigmentation—Light or amelanotic	1.43	0.61–3.34	0.40767
Tumor pigmentation—Moderate	1.35	0.67–2.72	0.40493
Baseline BCVA—<0.5 and >0.1	1.01	0.57–1.77	0.98235
Baseline BCVA—≤0.1	1.01	0.52–1.98	0.97040
**Retinopathy**			
**Age (years)**	**0.98**	**0.97–1.00**	**0.03547**
Treatment method—PT	0.65	0.30–1.44	0.28936
Treatment method—Ru-106 CCB	0.58	0.29–1.16	0.12180
**Treatment method—Ru-106 COB**	**0.10**	**0.03–0.37**	**0.00065**
Systemic hypertension—Yes	0.69	0.48–1.01	0.05636
Tumor thickness (mm)	0.96	0.82–1.12	0.58750
Tumor largest base diameter (mm)	0.98	0.89–1.08	0.71993
**Tumor location—Anterior to the equator**	**0.34**	**0.17–0.68**	**0.00227**
Tumor location—Posterior to the equator without the macula and optic disc	0.86	0.54–1.35	0.49979
Tumor location—Equator	0.78	0.44–1.37	0.38374
Tumor shape—Domed	0.69	0.37–1.28	0.23678
Tumor shape—Flat	0.81	0.19–3.45	0.77207
Tumor pigmentation—Light or amelanotic	1.86	0.99–3.49	0.05343
Tumor pigmentation—Moderate	1.53	0.89–2.64	0.12370
Baseline BCVA—<0.5 and >0.1	1.18	0.75–1.86	0.46238
Baseline BCVA—≤0.1	1.33	0.76–2.32	0.32131
**Cataract**			
**Age (years)**	**1.03**	**1.01–1.04**	**0.00154**
Treatment method—PT	1.53	0.70–3.37	0.28941
Treatment method—Ru-106 CCB	1.10	0.55–2.22	0.78304
Treatment method—Ru-106 COB	1.77	0.74–4.22	0.19700
Tumor thickness (mm)	1.12	0.97–1.28	0.12090
Tumor largest base diameter (mm)	1.06	0.97–1.16	0.19963
Tumor shape—Domed	0.84	0.46–1.54	0.56641
Tumor shape—Flat	0.36	0.07–1.82	0.21961
Baseline BCVA—<0.5 and >0.1	1.39	0.91–2.13	0.13084
Baseline BCVA—≤0.1	1.68	1.00–2.82	0.05161

* Due to the insufficient number of cases identified, analyses for hyphema, retinal detachment, keratopathy and dry eye were abandoned. ** The following reference values were selected for qualitative variables: baseline BCVA—≥0.5, tumor pigmentation—heavy, tumor shape—mushroom-shaped, tumor location—macula and/or optic disc, systemic hypertension—no, sex—male, treatment method—I-125, affected eye—right.

**Table 8:** Total number of complications occurring in individual patients.

Number of Complications	Number of Patients
0	36 (12.00)
1	66 (22.00)
2	67 (22.33)
3	53 (17.67)
4	42 (14.00)
5	21 (7.00)
6	10 (3.33)
7	3 (1.00)
8	2 (0.67)

Variables presented as n (%).

## Data Availability

The authors declare that due to legal restrictions related to the protection of patients’ personal data and the fact that data are part of ongoing studies, it is not possible to make the created database available to other researchers or readers. We have presented as much detailed data as possible in the article text and tables. In special cases, please contact the corresponding author.

## Abbreviations

The following abbreviations are used in this manuscript:

UM	uveal melanoma
BT	brachytherapy
PT	proton therapy
SRT	stereotactic radiotherapy
SRS	stereotactic radiosurgery
TTT	transpupillary thermotherapy
PDT	photodynamic therapy
Ru-106	ruthenium-106
I-125	iodine-125
COMS	Collaborative Ocular Melanoma Study
IOL	intraocular lens
BCVA	best corrected visual acuity
USG	ultrasonography
UBM	ultrasound biomicroscopy
AJCC	American Joint Committee on Cancer
OCT	optical coherence tomography
CGE	Cobalt Gray Equivalent
Anti-VEGF	anti-vascular endothelial growth factor
TCA	triamcinolone acetonide
SD	standard deviation
Ru-106 CCB brachytherapy	ruthenium-106 brachytherapy with CCB plaque
Ru-106 COB brachytherapy	ruthenium-106 brachytherapy with COB plaque
Ru-106 CCB (in tables)	ruthenium-106 brachytherapy with CCB plaque
Ru-106 COB (in tables)	ruthenium-106 brachytherapy with COB plaque
I-125 (in tables)	iodine-125 brachytherapy
mm	millimeter
Gy	gray
HR	hazard ratio
CI	confidence interval
TBUT	tear break-up time

## References

[1] Romanowska-Dixon B., Jakubowska B., Karska-Basta I., Kubicka-Trząska A., Damato B., Romanowska-Dixon B., Jager M.J., Coupland S.E. (2019). Guzy naczyniówki. Onkologia Okulistyczna.

[2] Romanowska-Dixon B., Jakubowska B. (2014). Czerniak Błony Naczyniowej. Atlas. Diagnostyka Różnicowa Nowotworów Wewnątrzgałkowych.

[3] Buonanno F., Conson M., de Almeida Ribeiro C., Oliviero C., Itta F., Liuzzi R., Pacelli R., Cella L., Clemente S. (2022). Local tumor control and treatment related toxicity after plaque brachytherapy for uveal melanoma: A systematic review and a data pooled analysis. Radiother. Oncol..

[4] Jovanovic P., Mihajlovic M., Djordjevic-Jocic J., Vlajkovic S., Cekic S., Stefanovic V. (2013). Ocular melanoma: An overview of the current status. Int. J. Clin. Exp. Pathol..

[5] Markiewicz A., Donizy P., Nowak M., Krzyziński M., Elas M., Płonka P.M., Orłowska-Heitzmann J., Biecek P., Hoang M.P., Romanowska-Dixon B. (2022). Amelanotic Uveal Melanomas Evaluated by Indirect Ophthalmoscopy Reveal Better Long-Term Prognosis Than Pigmented Primary Tumours-A Single Centre Experience. Cancers.

[6] Jager M.J., Shields C.L., Cebulla C.M., Abdel-Rahman M.H., Grossniklaus H.E., Stern M.-H., Carvajal R.D., Belfort R.N., Jia R., Shields J.A. (2020). Uveal melanoma. Nat. Rev. Dis. Primers.

[7] Messineo D., Barile G., Morrone S., La Torre G., Turchetti P., Accetta L., Battagliola E.T., Agostinelli E., Pacella F. (2020). Meta-analysis on the utility of radiotherapy for the treatment of Ocular Melanoma. Clin. Ter..

[8] Reichstein D.A., Brock A.L. (2021). Radiation therapy for uveal melanoma: A review of treatment methods available in 2021. Curr. Opin. Ophthalmol..

[9] Rusňák Š., Hecová L., Kasl Z., Sobotová M., Hauer L. (2020). Therapy of uveal melanoma A Review. Cesk. Slov. Oftalmol..

[10] Lee S.M., Kim M. (2025). Surgical treatment of uveal melanoma: Exoresection versus endoresection. Taiwan J. Ophthalmol..

[11] Karimi S., Arabi A., Siavashpour Z., Shahraki T., Ansari I. (2021). Efficacy and complications of ruthenium-106 brachytherapy for uveal melanoma: A systematic review and meta-analysis. J. Contemp. Brachytherapy.

[12] Echegaray J.J., Bechrakis N.E., Singh N., Bellerive C., Singh A.D. (2017). Iodine-125 Brachytherapy for Uveal Melanoma: A Systematic Review of Radiation Dose. Ocul. Oncol. Pathol..

[13] Hérault J., Gérard A., Carnicer A., Aloi D., Peyrichon M.-L., Barnel C., Vidal M., Angellier G., Fayaud D., Grini J.-C. (2022). 30 years of ocular proton therapy, the Nice view. Cancer Radiother..

[14] Collaborative Ocular Melanoma Study Group (1998). The Collaborative Ocular Melanoma Study (COMS) randomized trial of pre-enucleation radiation of large choroidal melanoma II: Initial mortality findings. COMS report no. 10. Am. J. Ophthalmol..

[15] Diener-West M., Earle J.D., Fine S.L., Hawkins B.S., Moy C.S., Reynolds S.M., Schachat A.P., Straatsma B.R., Collaborative Ocular Melanoma Study Group (2001). The COMS randomized trial of iodine 125 brachytherapy for choroidal melanoma, III: Initial mortality findings. COMS Report No. 18. Arch. Ophthalmol..

[16] Margo C.E. (2004). The Collaborative Ocular Melanoma Study: An overview. Cancer Control.

[17] Zemba M., Dumitrescu O.M., Gheorghe A.G., Radu M., Ionescu M.A., Vatafu A., Dinu V. (2023). Ocular Complications of Radiotherapy in Uveal Melanoma. Cancers.

[18] Wilson M.W., Hungerford J.L. (1999). Comparison of episcleral plaque and proton beam radiation therapy for the treatment of choroidal melanoma. Ophthalmology.

[19] O’Day R.F.J., Roelofs K.A., Negretti G.S., Hay G., Arora A.K., Stoker I., Damato B.E., Sagoo M.S., Cohen V.M.L. (2023). Long-term visual outcomes after ruthenium plaque brachytherapy for posterior choroidal melanoma. Eye.

[20] Shields C.L., Shields J.A., Cater J., Gündüz K., Miyamoto C., Micaily B., Brady L.W. (2000). Plaque radiotherapy for uveal melanoma: Long-term visual outcome in 1106 consecutive patients. Arch. Ophthalmol..

[21] Jung S.K., Park Y.H., Shin D.H., Kim H.-S., Jung J.-H., Kim T.-H., Moon S.H. (2020). Visual outcomes of proton beam therapy for choroidal melanoma at a single institute in the Republic of Korea. PLoS ONE.

[22] Oare C., Sun S., Dusenbery K., Reynolds M., Koozekanani D., Gerbi B., Ferreira C. (2022). Analysis of dose to the macula, optic disc, and lens in relation to vision toxicities—A retrospective study using COMS eye plaques. Phys. Medica.

[23] Espensen C.A., Kiilgaard J.F., Appelt A.L., Fog L.S., Herault J., Maschi C., Caujolle J.-P., Thariat J. (2021). Dose-Response and Normal Tissue Complication Probabilities after Proton Therapy for Choroidal Melanoma. Ophthalmology.

[24] Marinkovic M., Pors L.J., van den Berg V., Peters F.P., Schalenbourg A., Zografos L., Pica A., Hrbacek J., Van Duinen S.G., Vu T.H.K. (2021). Clinical Outcomes after International Referral of Uveal Melanoma Patients for Proton Therapy. Cancers.

[25] Hasegawa N., Teh B.S., Tran K., Ivey F., Olek D., Pino R., Chuang A.Z., Bretana M.E., Butler E.B., Schefler A.C. (2024). Retrospective Analysis of Radiation-Induced Complications of Uveal Melanoma Patients Treated with Brachytherapy in the Era of Anti-VEGF. Am. J. Ophthalmol..

[26] Tseng V., Coleman A., Zhang Z., McCannel T. (2016). Complications from Plaque versus Proton Beam Therapy for Choroidal Melanoma: A Qualitative Systematic Review. J. Cancer Ther..

[27] Chang M., Dalvin L.A., Mazloumi M., Martin A., Yaghy A., Yang X., Bakhtiari S.B., Li L.B., Jennings E.B., Mashayekhi A. (2020). Prophylactic Intravitreal Bevacizumab After Plaque Radiotherapy for Uveal Melanoma: Analysis of Visual Acuity, Tumor Response, and Radiation Complications in 1131 Eyes Based on Patient Age. Asia Pac. J. Ophthalmol..

[28] Puusaari I., Heikkonen J., Kivelä T. (2004). Ocular complications after iodine brachytherapy for large uveal melanomas. Ophthalmology.

[29] Dogrusöz M., Jager M.J., Damato B. (2017). Uveal Melanoma Treatment and Prognostication. Asia Pac. J. Ophthalmol..

[30] Pors L.J., Marinkovic M., Deuzeman H.H., Vu T., Kerkhof E., van Wieringen-Warmenhoven K., Rasch C., Bleeker J., Koetsier L., Beenakker J. (2025). Clinical outcomes and risk factors for local failure and visual impairment in patients treated with Ru-106 brachytherapy for uveal melanoma. Clin. Transl. Radiat. Oncol..

[31] Cennamo G., Montorio D., D’ Andrea L., Farella A., Matano E., Giuliano M., Liuzzi R., Breve M.A., De Placido S., Cennamo G. (2022). Long-Term Outcomes in Uveal Melanoma After Ruthenium-106 Brachytherapy. Front. Oncol..

[32] Mirshahi R., Sedaghat A., Jaberi R., Azma Z., Mazloumi M., Naseripour M. (2022). Ruthenium-106 plaque radiotherapy for uveal melanoma: Analysis of tumor dimension and location on anatomical and functional results. BMC Ophthalmol..

[33] Ghassemi F., Sheibani S., Arjmand M., Poorbaygi H., Kouhestani E., Sabour S., Samiei F., Beiki-Ardakani A., Jabarvand M., Tari A.S. (2020). Comparison of Iodide-125 and Ruthenium-106 Brachytherapy in the Treatment of Choroidal Melanomas. Clin. Ophthalmol..

[34] Seddon J.M., Gragoudas E.S., Polivogianis L., Hsieh C.-C., Egan K.M., Goitein M., Verhey L., Munzenrider J., Austin-Seymour M., Urie M. (1986). Visual outcome after proton beam irradiation of uveal melanoma. Ophthalmology.

[35] Guleser U.Y., Sarici A.M., Ucar D., Gonen B., Sengul Samanci N., Özgüroğlu M. (2022). Comparison of iodine-125 plaque brachytherapy and gamma knife stereotactic radiosurgery treatment outcomes for uveal melanoma patients. Graefes Arch. Clin. Exp. Ophthalmol..

[36] Bolling J.P., Dagan R., Rutenberg M., Mamalui-Hunter M., Buskirk S.J., Heckman M.G., Hochwald A.P., Slopsema R. (2021). Treatment of Uveal Melanoma with Radioactive Iodine 125 Implant Compared with Proton Beam Radiotherapy. Mayo Clin. Proc. Innov. Qual. Outcomes.

[37] Char D.H., Quivey J.M., Castro J.R., Kroll S., Phillips T. (1993). Helium ions versus iodine 125 brachytherapy in the management of uveal melanoma. A prospective, randomized, dynamically balanced trial. Ophthalmology.

[38] Shields C.L., Cater J., Shields J.A., Chao A., Krema H., Materin M., Brady L.W. (2002). Combined plaque radiotherapy and transpupillary thermotherapy for choroidal melanoma: Tumor control and treatment complications in 270 consecutive patients. Arch. Ophthalmol..

[39] Damato B., Patel I., Campbell I.R., Mayles H.M., Errington R.D. (2005). Visual acuity after Ruthenium(106) brachytherapy of choroidal melanomas. Int. J. Radiat. Oncol. Biol. Phys..

[40] Marinkovic M., Horeweg N., Fiocco M., Peters F.P., Sommers L.W., Laman M.S., Bleeker J.C., Ketelaars M., Luyten G.P., Creutzberg C.L. (2016). Ruthenium-106 brachytherapy for choroidal melanoma without transpupillary thermotherapy: Similar efficacy with improved visual outcome. Eur. J. Cancer.

[41] Espensen C.A., Appelt A.L., Fog L.S., Gothelf A.B., Thariat J., Kiilgaard J.F. (2019). Predicting Visual Acuity Deterioration and Radiation-Induced Toxicities after Brachytherapy for Choroidal Melanomas. Cancers.

[42] Patel A.V., Lane A.M., Morrison M.A., Trofimov A.V., Shih H.A., Gragoudas E.S., Kim I.K. (2016). Visual Outcomes after Proton Beam Irradiation for Choroidal Melanomas Involving the Fovea. Ophthalmology.

[43] Aziz H.A., Singh N., Bena J., Wilkinson A., Singh A.D. (2016). Vision Loss Following Episcleral Brachytherapy for Uveal Melanoma: Development of a Vision Prognostication Tool. JAMA Ophthalmol..

[44] Mourtada F., Koch N., Newhauser W. (2005). 106Ru/106Rh plaque and proton radiotherapy for ocular melanoma: A comparative dosimetric study. Radiat Prot. Dosim..

[45] Jasani A., Norris L., McGwin G., Mason J.O. (2025). Up to ten years of visual acuity outcomes in fellow eyes post-uveal melanoma treatment with iodine-125 radiotherapy, transpupillary thermotherapy, and proton beam therapy. J. Contemp. Brachytherapy.

[46] Stoffelns B.M., Kutzner J., Schöpfer K., Frising M. (2002). Prospektive nicht randomisierte Analyse der “Sandwich-Therapie” beim malignen Melanom der Aderhaut [Prospective nonrandomised analysis of “Sandwich Therapy” for malignant melanoma of the choroid]. Klin. Monatsblatter fur Augenheilkd..

[47] Kivelä T., Simpson E.R., Grossniklaus H.E., Jager M.J., Singh A.D., Caminal J.M., Pavlick A.C., Kujala E., Coupland S.E., Finger P.T., Amin M.B., Edge S., Greene F., Byrd D.R., Brookland R.K., Washington M.K., Gershenwald J.E., Compton C.C., Hess K.R., Sullivan D.C. (2017). Chapter 67: Uveal Melanoma. Ophthalmic Sites: Part XV. AJCC Cancer Staging Manual.

[48] The Collaborative Ocular Melanoma Study Group (1997). Mortality in patients with small choroidal melanoma. COMS report no. 4. Arch. Ophthalmol..

[49] Sas-Korczyńska B., Markiewicz A., Romanowska-Dixon B., Pluta E. (2014). Preliminary results of proton radiotherapy for choroidal melanoma—The Kraków experience. Contemp. Oncol..

[50] Romanowska-Dixon B., Dębicka-Kumela M., Śmigielski J., Nowak M.S. (2023). Sex Differences in the Treatment of Uveal Melanoma in a Group of 1336 Patients. J. Pers. Med..

[51] Kivelä T., Eskelin S., Mäkitie T., Summanen P. (2001). Exudative retinal detachment from malignant uveal melanoma: Predictors and prognostic significance. Investig. Ophthalmol. Vis. Sci..

[52] Seibel I., Cordini D., Hager A., Riechardt A.I., Rehak M., Böker A., Böhmer D., Heufelder J., Joussen A.M. (2016). Cataract development in patients treated with proton beam therapy for uveal melanoma. Graefes Arch. Clin. Exp. Ophthalmol..

[53] Summanen P., Immonen I., Kivelä T., Tommila P., Heikkonen J., Tarkkanen A. (1996). Radiation related complications after ruthenium plaque radiotherapy of uveal melanoma. Br. J. Ophthalmol..

[54] Tarmann L., Wackernagel W., Ivastinovic D., Schneider M., Winkler P., Langmann G. (2017). Tumor parameters predict the risk of side effects after ruthenium-106 plaque brachytherapy of uveal melanomas. PLoS ONE.

[55] Seibel I., Cordini D., Hager A., Tillner J., Riechardt A.I., Heufelder J., Davids A.M., Rehak M., Joussen A.M. (2016). Predictive risk factors for radiation retinopathy and optic neuropathy after proton beam therapy for uveal melanoma. Graefes Arch. Clin. Exp. Ophthalmol..

[56] Pagliara M.M., Tagliaferri L., Azario L., Lenkowicz J., Lanza A., Autorino R., Caputo C.G., Gambacorta M.A., Valentini V., Blasi M.A. (2018). Ruthenium brachytherapy for uveal melanomas: Factors affecting the development of radiation complications. Brachytherapy.

[57] Schönfeld S., Cordini D., Riechardt A.I., Seibel I., Willerding G., Bechrakis N.E., Moser L., Joussen A.M. (2014). Proton beam therapy leads to excellent local control rates in choroidal melanoma in the intermediate fundus zone. Am. J. Ophthalmol..

[58] Puusaari I., Heikkonen J., Kivelä T. (2004). Effect of radiation dose on ocular complications after iodine brachytherapy for large uveal melanoma: Empirical data and simulation of collimating plaques. Investig. Ophthalmol. Vis. Sci..

[59] Shen L., Melles R.B., Metlapally R., Barcellos L., Schaefer C., Risch N., Herrinton L.J., Wildsoet C., Jorgenson E. (2016). The Association of Refractive Error with Glaucoma in a Multiethnic Population. Ophthalmology.

[60] Marcus M.W., de Vries M.M., Junoy Montolio F.G., Jansonius N.M. (2011). Myopia as a risk factor for open-angle glaucoma: A systematic review and meta-analysis. Ophthalmology.

[61] Conway R.M., Poothullil A.M., Daftari I.K., Weinberg V., Chung J.E., O’Brien J.M. (2006). Estimates of ocular and visual retention following treatment of extra-large uveal melanomas by proton beam radiotherapy. Arch. Ophthalmol..

[62] Quivey J.M., Char D.H., Phillips T.L., Weaver K.A., Castro J.R., Kroll S.M. (1993). High intensity 125-iodine (125I) plaque treatment of uveal melanoma. Int. J. Radiat. Oncol. Biol. Phys..

[63] Thariat J., Maschi C., Lanteri S., Peyrichon M.L., Baillif S., Herault J., Salleron J., Caujolle J.P. (2017). Dry Eye Syndrome After Proton Therapy of Ocular Melanomas. Int. J. Radiat. Oncol. Biol. Phys..

[64] Gill V.T., Stålhammar G. (2023). Incidence, risk factors and outcomes of cataract surgery after plaque brachytherapy for posterior uveal melanoma. Heliyon.

[65] Shields C.L., Dalvin L.A., Chang M., Mazloumi M., Fortin P., McGarrey M., Martin A., Yaghy A., Yang X., Vichitvejpaisal P. (2020). Visual Outcome at 4 Years Following Plaque Radiotherapy and Prophylactic Intravitreal Bevacizumab (Every 4 Months for 2 Years) for Uveal Melanoma: Comparison with Nonrandomized Historical Control Individuals. JAMA Ophthalmol..

[66] Jarczak J., Karska-Basta I., Romanowska-Dixon B. (2023). Deterioration of Visual Acuity after Brachytherapy and Proton Therapy of Uveal Melanoma, and Methods of Counteracting This Complication Based on Recent Publications. Medicina.

[67] Stålhammar G. (2023). Brachytherapy with 15- Versus 20-mm Ruthenium 106 Plaques without Verification of Plaque Position Is Associated with Local Tumor Recurrence and Death in Posterior Uveal Melanoma. Int. J. Radiat. Oncol. Biol. Phys..

[68] Damato B., Eleuteri A., Coupland S.E., Kalirai H., Heimann H. (2025). How Many Patients with Choroidal Melanoma Would Be Eligible for Neoadjuvant Systemic Therapy to Enable Ruthenium-106 Brachytherapy?. Cancers.

[69] Hiong A., O’Day R., Fog L.S., McKay D., McKenzie J., Ameratunga M., Joshua A.M., Shackleton M. (2024). Globe Salvage and Vision Preservation by Neoadjuvant Darovasertib and Crizotinib in Uveal Melanoma. Ophthalmol. Retina.

[70] Śniegocka M., Podgórska E., Płonka P.M., Elas M., Romanowska-Dixon B., Szczygieł M., Żmijewski M.A., Cichorek M., Markiewicz A., Brożyna A.A. (2018). Transplantable Melanomas in Hamsters and Gerbils as Models for Human Melanoma. Sensitization in Melanoma Radiotherapy-From Animal Models to Clinical Trials. Int. J. Mol. Sci..

[71] Zhang B., Wu H., Hao J., Wu Y., Yang B. (2020). Inhibition of DNA-PKcs activity re-sensitizes uveal melanoma cells to radio- and chemotherapy. Biochem. Biophys. Res. Commun..

